# Endurance exercise training-responsive miR-19b-3p improves skeletal muscle glucose metabolism

**DOI:** 10.1038/s41467-021-26095-0

**Published:** 2021-10-12

**Authors:** Julie Massart, Rasmus J. O. Sjögren, Brendan Egan, Christian Garde, Magnus Lindgren, Weifeng Gu, Duarte M. S. Ferreira, Mutsumi Katayama, Jorge L. Ruas, Romain Barrès, Donal J. O’Gorman, Juleen R. Zierath, Anna Krook

**Affiliations:** 1 Department of Molecular Medicine and Surgery, Section of Integrative Physiology, Karolinska Institutet, Stockholm, Sweden; 2 Department of Physiology and Pharmacology, Section of Integrative Physiology, Karolinska Institutet, Stockholm, Sweden; 3 Department of Health & Human Performance, Dublin City University, Dublin, Ireland; 4 Novo Nordisk Foundation Center for Basic Metabolic Research, University of Copenhagen, Copenhagen, Denmark; 5 University of Massachusetts Medical School, Worchester, MA USA; 6 Department of Physiology and Pharmacology, Section of Molecular and Cellular Exercise Physiology, Karolinska Institutet, Stockholm, Sweden; 7 Present Address: Department of Cell Biology and Neuroscience, University of California at Riverside, Riverside, CA USA

**Keywords:** miRNAs, Endocrine system and metabolic diseases, Molecular medicine

## Abstract

Skeletal muscle is a highly adaptable tissue and remodels in response to exercise training. Using short RNA sequencing, we determine the miRNA profile of skeletal muscle from healthy male volunteers before and after a 14-day aerobic exercise training regime. Among the exercise training-responsive miRNAs identified, miR-19b-3p was selected for further validation. Overexpression of miR-19b-3p in human skeletal muscle cells increases insulin signaling, glucose uptake, and maximal oxygen consumption, recapitulating the adaptive response to aerobic exercise training. Overexpression of miR-19b-3p in mouse flexor digitorum brevis muscle enhances contraction-induced glucose uptake, indicating that miR-19b-3p exerts control on exercise training-induced adaptations in skeletal muscle. Potential targets of miR-19b-3p that are reduced after aerobic exercise training include *KIF13A*, *MAPK6*, *RNF11*, and *VPS37A*. Amongst these, RNF11 silencing potentiates glucose uptake in human skeletal muscle cells. Collectively, we identify miR-19b-3p as an aerobic exercise training-induced miRNA that regulates skeletal muscle glucose metabolism.

## Abstract (web summary)

Exercise induces structural and functional adaptations in skeletal muscle that involve transcriptomic remodeling, including of miRNA expression. Here the authors examine the expression of miRNAs in human muscle following exercise training and investigate the functions of miR-19b-3p on glucose metabolism in cells and mouse muscle.

## Introduction

The benefits of exercise are pleiotropic, including systemic effects on cardiac function, energetics, and the vascular system, as well as intrinsic effects in working skeletal muscle including substrate metabolism and changes in contractile proteins and function^1^. Aerobic exercise training augments whole-body insulin sensitivity and improves glucose tolerance in healthy and insulin-resistant individuals^2–4^, thereby reducing the risk of developing type 2 diabetes^5^. While exercise training is recommended to alleviate insulin resistance in type 2 diabetic patients^6^, many of the molecular transducers of the effects of exercise in skeletal muscle remain to be identified.

Exercise-induced adaptations (i.e., increased mitochondrial density, altered substrate metabolism, or myofiber hypertrophy) in skeletal muscle are mediated by signaling events, pre- and post-transcriptional processes, regulation of translation, and ultimately, increased abundance and/or activity of proteins regulating these cellular processes^7^. Gene expression and protein abundance in skeletal muscle are robustly altered by aerobic exercise training^7,8^. These alterations are controlled by transcriptional networks involving numerous transcriptional regulators^7^. Transcription factors and epigenetic changes are candidate factors for such regulatory events. Mechanistic insight into the specific molecular and cellular events by which exercise enhances insulin sensitivity and preserves muscle mass may uncover novel therapeutic entry points to mimic or enhance exercise response in individuals unable to partake in training programs.

microRNAs (miRNAs) are a class of short, non-coding RNAs that regulate gene expression by reducing protein abundance of targeted genes^9^. miRNAs guide the RNA-induced silencing complex (RISC) to targeted mRNAs with 3′ UTR complementary sites, ultimately leading to reduced protein translation of miRNA targets. Aerobic exercise training remodels miRNA expression in skeletal muscle^8,10^. Several miRNAs are altered in response to acute aerobic exercise^11^, indicating the potential of miRNA-dependent regulation of skeletal muscle exercise adaptation. However, the direct role of specific miRNAs in the regulation of gene expression and metabolism in skeletal muscle in response to exercise training remains unknown. Here we performed unbiased short RNA sequencing to determine the miRNA profile of human skeletal muscle and the adaptive response to aerobic exercise training. We hypothesized that miRNAs with altered expression following aerobic exercise training influence insulin sensitivity and energy substrate metabolism in skeletal muscle.

## Results

### Aerobic exercise training regulates multiple miRNAs in human skeletal muscle

The potential to repress target genes is considered to be mainly reserved for miRNAs with high cellular abundance^12^. Thus, we performed short RNA sequencing to determine miRNAs expression levels in a subset of human skeletal muscle biopsies collected before and after an endurance exercise training program (*n* = 3 subjects). We identified 102 miRNAs with an expression level higher than 100 reads per million (RPM) in at least 50% of the biopsies (Supplementary Table 1). These 102 miRNAs represent skeletal muscle miRNAs with potential repressive activity either at rest, or in response to exercise training. Amongst these miRNAs, 93 had a mean expression above 100 RPM in biopsies collected before exercise training. An additional nine miRNAs had mean expression above 100 RPM in biopsies collected after exercise training. Several muscle-enriched miRNAs (myomiRs) including miR-1-3p, miR-133a-3p, and miR-133b, were amongst the identified miRNAs with more than 10,000 RPM in biopsies collected before exercise training (Table 1). This analysis reveals the miRNA expression profile of human skeletal muscle.

**Table 1:** Most abundant miRNAs in human skeletal muscle biopsies.

miRNA	RPM
hsa-miR-1-3p	208,397 ± 61,354
hsa-miR-133a-3p	37,577 ± 16,582
hsa-let-7c-5p	31, 870 ± 13,260
hsa-miR-133b	31,238 ± 15,654
hsa-let-7b-5p	31,047 ± 12,910
hsa-let-7a-5p	29,562 ± 12,453
hsa-let-7f-5p	21,434 ± 8186
hsa-miR-29c-3p	19,759 ± 5915
hsa-miR-451a	18,272 ± 7608
hsa-miR-206	12,371 ± 1978
hsa-miR-23b-3p	11,948 ± 823

Short RNA sequencing of human skeletal muscle biopsies obtained before exercise training was performed to determine miRNA abundance (reads per million (RPM) > 10,000). Results are mean ± SEM for three subjects.

**Fig. 1:**
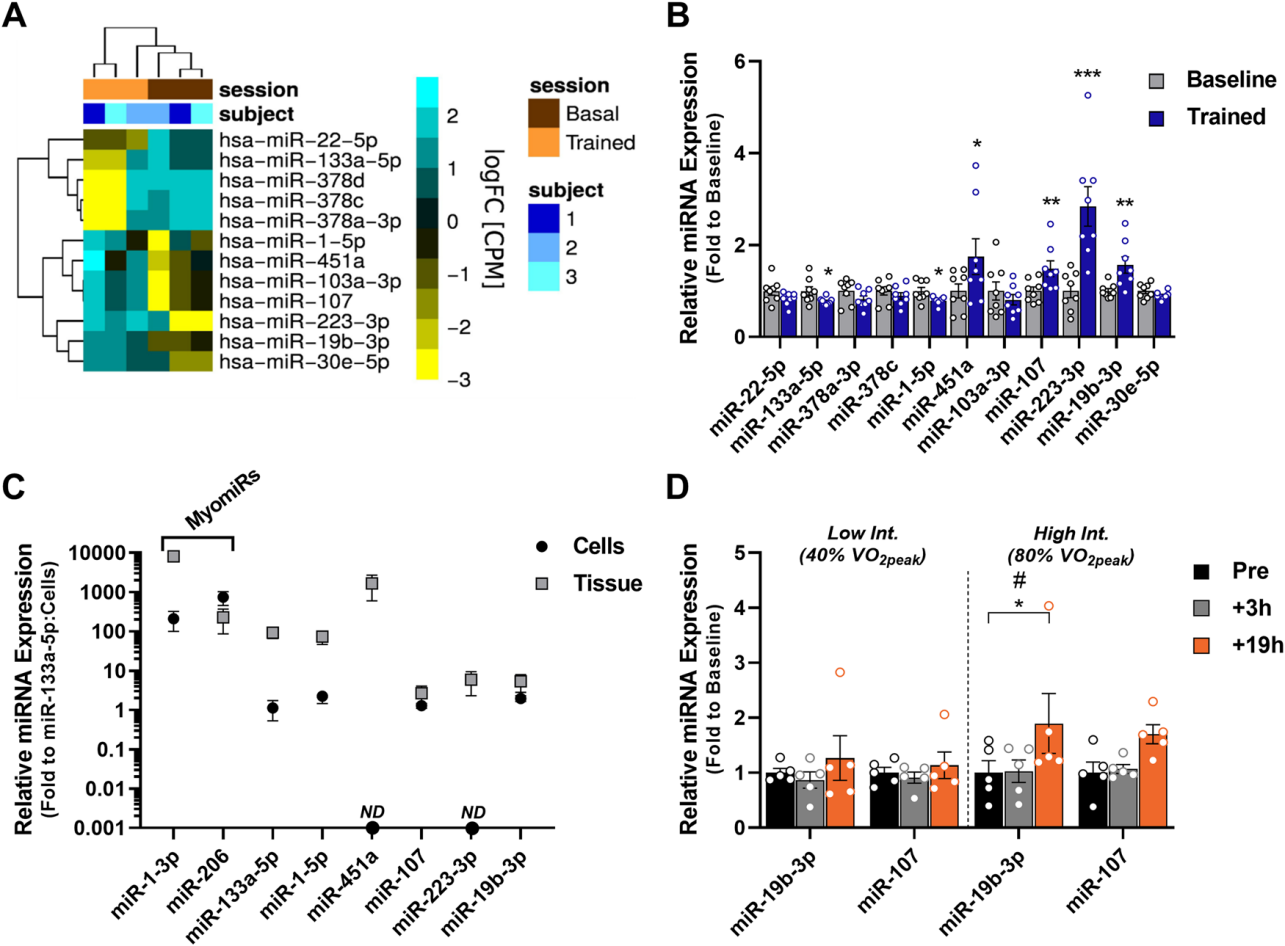
Aerobic exercise training regulates multiple miRNAs in human skeletal muscle. Skeletal muscle biopsies were obtained from healthy volunteers before and after an aerobic exercise training program. **A** Altered miRNAs as determined by short RNA sequencing (*n* = 3 individuals; FDR < 0.05). **B** RT-qPCR determination of miRNA expression before (Baseline) and after 14-day aerobic exercise training (Trained) of miRNAs identified by short RNA sequencing shown in (**A**) (*n* = 8 individuals) (*p* = 0.024 for miR-133a-5p; *p* = 0.0236 for miR-1-5p; *p* = 0.0224 for miR-451a; *p* = 0.0029 for miR-107; *p* = 0.0008 for miR-223.3p; *p* = 0.0013 for miR-19b-3p). **C** Relative miRNA expression in human skeletal muscle biopsies versus myotubes differentiated in vitro (data are mean ± SD for *n* = 3 cell culture independent samples and *n* = 8 tissue independent samples). **D** miR-19b-3p and miR-107 expression determined by RT-qPCR in human skeletal muscle biopsies obtained at baseline, 3 h, and 19 h after either low intensity (40% VO_2peak_) or high intensity (80% VO_2peak_) aerobic exercise (*p* = 0.0228 for miR-19b-3p 19 h following high-intensity exercise; *n* = 5 individuals). miRNA RT-qPCR data were normalized to miR-186-5p. Data are mean ± SEM. # Exercise effect (Friedman’s test); *p < 0.05, **p < 0.01, ***p < 0.001 using two-tailed paired Student’s *t*-test or Friedman’s test followed by Dunn’s multiple comparison test.

Short RNA sequencing identified 12 miRNAs with differential expression in skeletal muscle after aerobic exercise training (Fig. 1A). In the absence of a specific probe for miR-378d, 11 of these 12 miRNAs were subjected to a secondary validation by RT-qPCR in skeletal muscle from eight volunteers, including the 3 subjects from the RNA sequencing analysis and 5 additional subjects undergoing the same aerobic exercise training program. RT-qPCR results were highly correlated with the short RNA sequencing data (Supplementary Fig. 1A). We found that 6 out of the 11 miRNAs showed altered abundance (Fig. 1B). The relatively less abundant myomiRs miR-1-5p and miR-133a-5p showed decreased expression following exercise training (Fig. 1B), consistent with a trend for decreased expression of other myomiRs (Supplementary Fig. 1B). We also identified increased expression of miR-19b-3p, miR-107, miR-223-3p, and miR-451a following exercise training (Fig. 1B). Skeletal muscle biopsies mainly consist of myocytes, but other cell types such as skeletal muscle progenitors (satellite cells), endothelial cells, and various circulating blood cells are present. To identify whether these miRNAs are expressed in myocytes, we determined their abundance in cultured human primary myotubes and compared these results with the abundance in skeletal muscle biopsies (Fig. 1C). We found miR-19b-3p and miR-107 are expressed to a similar extent in differentiated myotubes and biopsies, while miR-451a and miR-223-3p were undetectable in primary human myotubes (Fig. 1C). Most of the exercise training-regulated miRNAs identified in human skeletal muscle biopsies were also expressed in mouse soleus skeletal muscle even after removal of blood following cardiac perfusion (Supplementary Fig. 1C), with the exception of miR-451a, which showed expression to a similar degree as hemoglobin beta expression (Supplementary Fig. 1D), indicating that the primary source of miR-451a is of non-muscle origin. Given that miR-223-3p and miR-451a were not expressed in primary human skeletal muscle cells, the exercise-induced increase of these miRNAs is likely due to increased vascularization or infiltration of other cell types within the organ.

To gain further insight into the regulation of miR-19b-3p and miR-107 following exercise, we measured miR-19b-3p and miR-107 expression at different timepoints throughout the training period. We found a time-dependent effect of the exercise training regimen to alter miR-19b-3p and miR-107 expression, with a significant increase after 14 days (Supplementary Fig. 1E, F). In a subset of five individuals undergoing the same aerobic exercise training program, we found increased expression of both miR-19b-3p and miR-107 16 h after the first exercise bout of the training regimen (Supplementary Fig. 1G, H). miR-19b-1-3p is part of the miR-17/92 cluster, which includes miR-20a-5p and miR-92a-3p. We therefore examined the expression of miR-20a-5p and miR-92a-3p by RT-qPCR. miR-20a-5p was increased, while miR-92a-3p expression tended to increase (Supplementary Fig. 1I), suggesting transcriptional regulation of the cluster following exercise. To determine if exercise intensity regulates miR-19b-3p and miR-107 expression, we measured the levels of these miRNAs in skeletal muscle from subjects undergoing one bout of aerobic exercise at low (40% VO_2peak_) or high (80% VO_2peak_) intensity, at baseline, 3 h, and 19 h after each respective exercise session. While expression of miR-19b-3p and miR-107 was unaffected by low-intensity exercise at every time point, expression of miR-19b-3p was increased at 19 h, and miR-107 expression tended to be increased at 19 h after high-intensity exercise (*p* = 0.08) (Fig. 1D). Thus, transcriptional regulation of these two miRNAs occurs already at 19 h following moderate to high-intensity exercise. In addition, in mouse skeletal muscle, miR-19b-3p and miR-107 expression is higher in oxidative soleus muscle compared to glycolytic EDL muscle (Supplementary Fig. 1J). Since oxidative fibers are more recruited during endurance exercise compared to glycolytic fibers, these results are consistent with the increased expression following endurance exercise training. Thus, our analysis identifies miR-19b-3p and miR-107 as two miRNAs that are responsive to endurance exercise in human skeletal muscle.

### miR-19b-3p improves skeletal muscle glucose metabolism and insulin signaling

**Fig. 2:**
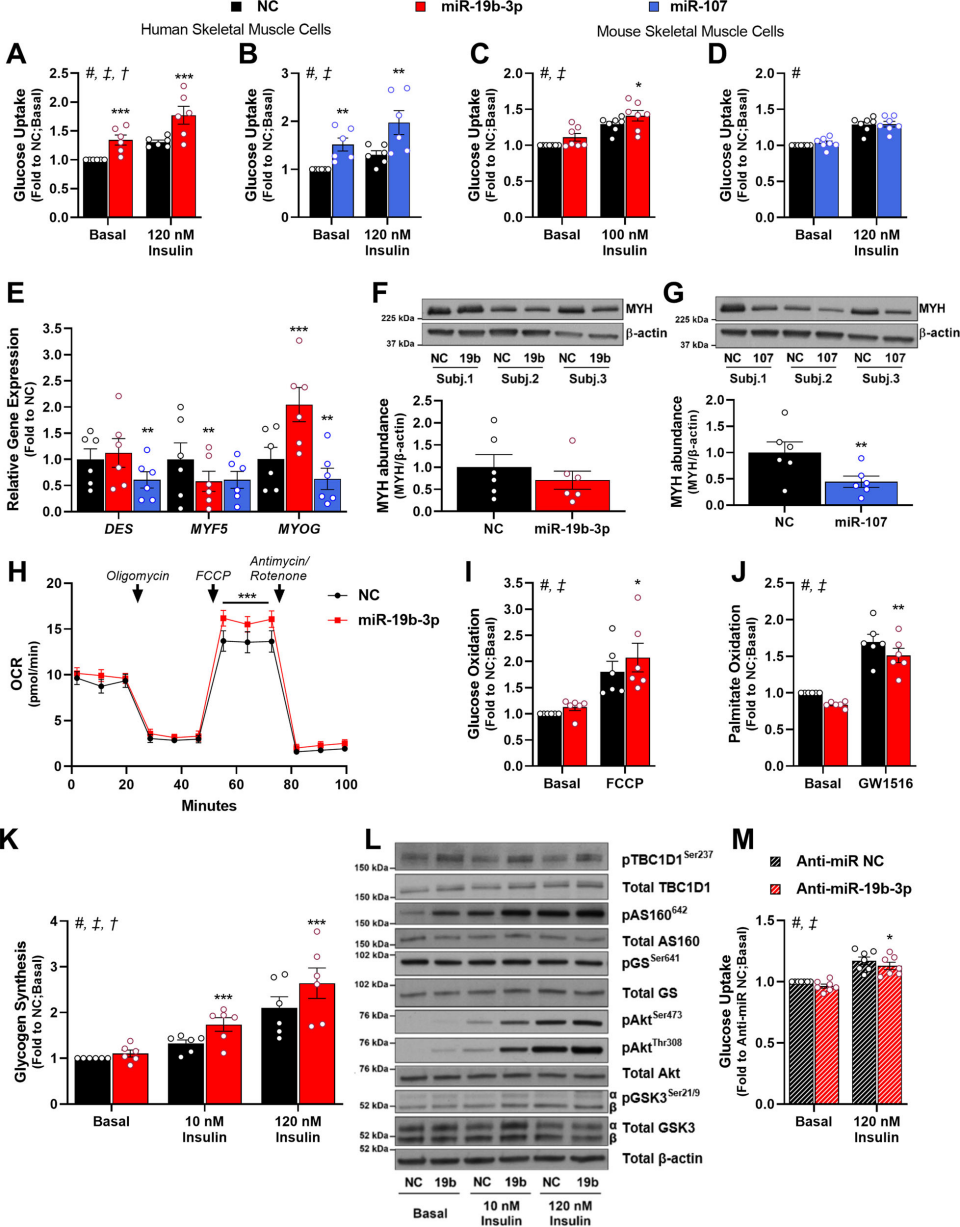
miR-19b-3p improves glucose metabolism and insulin sensitivity in skeletal muscle. Myotubes were transfected with miR-19b-3p or miR-107 precursors or a negative control (NC) miRNA precursor. **A**, **B** Human skeletal muscle myotubes were incubated in the absence (Basal) or presence of insulin (120 nM) to determine the effect of A) miR-19b-3p (*p* = 0.0003 for basal and *p* = 0.0001 for insulin) and **B** miR-107 overexpression (*p* = 0.0019 for basal and *p* = 0.0014 for insulin) on glucose uptake (*n* = 6 independent cell experiments). **C**, **D** Mouse skeletal muscle myotubes were incubated in the absence (Basal) or presence of insulin (100 nM) to determine effects of **C** miR-19b-3p (*p* = 0.0307 for insulin) and **D** miR-107 overexpression on glucose uptake (*n* = 7 independent cell experiments). **E** Expression of *DES*, *MYF5*, and *MYOG* was determined by RT-qPCR in human myotubes overexpressing either miR-19b-3p or miR-107 (*n* = 6 independent cell experiments). Data for human skeletal muscle cells were normalized to the geometrical mean of *GUSB* and *TBP* expression (miR-19b-3p: *p* = 0.0035 for *MYF5*; *p* = 0.0008 for *MYOG*; miR-107: *p* = 0.001 for *DES*; *p* = 0.0057 for *MYOG)*. **F**, **G** Western blot analysis and quantification of Myosin Heavy Chain 1 and 2 (MYH) abundance in human myotubes overexpressing **F** miR-19b-3p and **G** miR-107 (miR-107: *p* = 0.0067) (*n* = 6 independent cell experiments). **H** Oxygen Consumption Rate (OCR) in human myotubes overexpressing miR-19b-3p (*n* = 7 independent cell experiments). Addition of chemical substrates are indicated by arrows. *p* = 0.0001 following addition of FCCP. **I** Human myotubes were incubated in DMSO alone (Basal) or in the presence of 1 µM of the mitochondrial uncoupler FCCP to determine the effects of miR-19b-3p overexpression on glucose oxidation (*n* = 6 independent cell experiments) (*p* = 0.0241 for FCCP). **J** Human myotubes were incubated in DMSO alone (Basal) or in the presence of 10 nM GW1516 (PPARδ agonist) for 96 h prior to determination of miR-19b-3p overexpression on palmitate oxidation (*n* = 6 independent cell experiments) (*p* = 0.0026 for basal and *p* = 0.0013 for GW1516). **K** Human myotubes were incubated in the absence (Basal) or presence of insulin (10 or 120 nM) to determine the effect of miR-19b-3p overexpression on glucose incorporation into glycogen (*n* = 6 independent cell experiments) (*p* = 0.0009 for 10 nM insulin and *p* = 0.0001 for 120 nM insulin). **L** Representative immunoblots of human myotubes incubated for 10 min in the absence (Basal) or presence of insulin (10 or 120 nM) to determine the effects of miR-19b-3p overexpression on protein phosphorylation and protein abundance. Data of immunoblot quantification are found in Table S2. **M** Human myotubes were transfected with a miR-19b-3p-specific inhibitor (Anti-miR-19b-3p) or a negative control inhibitor (Anti-miR NC) and incubated in the absence (Basal) or presence of insulin (120 nM) to determine the effects of miR-19b-3p inhibition on glucose uptake (*n* = 7). Data are mean ± SEM. # Treatment effect, ‡ miRNA effect, † Interaction; and *p < 0.05, **p < 0.01, ***p < 0.001 by paired Student’s *t*-test or repeated measures 2-way ANOVA with Sidak’s post hoc testing when appropriate.

To determine the effects of miR-19b-3p and miR-107 on skeletal muscle glucose uptake, we overexpressed these miRNAs in differentiated human myotubes (Supplementary Fig. 2A, B). Basal and insulin-stimulated glucose uptake was increased following miR-19b-3p or miR-107 overexpression (Fig. 2A, B). In primary mouse skeletal muscle cells, miR-19b-3p overexpression enhanced insulin-stimulated glucose uptake (Fig. 2C and Supplementary Fig. 2C), while miR-107 overexpression was without effect (Fig. 2D and Supplementary Fig. 2D). Similarly, miR-19b-3p, but not miR-107, altered glucose uptake in C2C12 skeletal muscle cells (Supplementary Fig. 2E–H). To determine whether altered myotube differentiation accounts for changes in metabolism, we measured the expression of genes involved in skeletal muscle differentiation (Fig. 2E). In primary mouse skeletal muscle cells or in C2C12, miR-19b-3p, or miR-107 overexpression did not alter the expression of Desmin (DES) or the transcription factor governing muscle development Myogenin (MYOG) (Supplementary Fig. 2I, J). In human myotubes, miR-19b-3p overexpression decreased the expression of the proliferative marker Myogenic Factor 5 (MYF5) and increased the expression of MYOG (Fig. 2E), and was without effect on myosin heavy chain 1 and 2 (MYH1/2) protein abundance (Fig. 2F). Conversely, miR-107 overexpression decreased the expression of DES and MYOG and reduced MYH1/2 protein abundance in human skeletal muscle cells (Fig. 2E, G). These results indicate that miR-107 overexpression impairs human skeletal muscle cell differentiation. Given that the skeletal muscle differentiation status affects metabolism^13^, we further validated miR-19b-3p for a role on skeletal muscle metabolism.

Overexpression of miR-19b-3p improved maximal (FCCP-stimulated) oxygen consumption rate (OCR) in human skeletal muscle cells (Fig. 2H), indicative of increased reserve respiratory capacity. Following miR-19b-3p overexpression, glucose oxidation was increased (Fig. 2I), while palmitate oxidation was decreased (Fig. 2J), indicating a shift towards glucose oxidation. Insulin sensitivity and responsiveness of glycogen synthesis were improved following miR-19b-3p overexpression (Fig. 2K). In primary mouse skeletal muscle cells, miR-19b-3p overexpression increased basal and insulin-stimulated glycogen synthesis (Supplementary Fig. 2K). In addition, miR-19b-3p increased phosphorylation of Akt at Ser^473^ and Thr^308^ at a submaximal insulin dose (10 nM), with concomitant increased phosphorylation of downstream Akt targets such as AS160 (Thr^642^) and GSK3α (Ser^21^) (Fig. 2L and Supplementary Table 2). Overexpression of miR-19b-3p also increased phosphorylation of TBC1 Domain Family Member 1 (TBC1D1) at Ser^237^ with a concomitant increase in total TBC1D1 protein abundance (Fig. 2L and Supplementary Table 2). To determine the effects of endogenous miR-19b-3p on glucose metabolism, we utilized miRNA inhibitors in human myotubes to reduce miR-19b-3p availability (Supplementary Fig. 2L). Inhibition of miR-19b-3p reduced glucose uptake (Fig. 2M), without affecting basal or insulin-stimulated glycogen synthesis (Supplementary Fig. 2M). Collectively, these results indicate that miR-19b-3p overexpression recapitulates exercise training-associated effects on skeletal muscle, including improved insulin sensitivity and glucose metabolism.

### miR-19b-3p improves contraction-stimulated glucose transport in mouse skeletal muscle

**Fig. 3:**
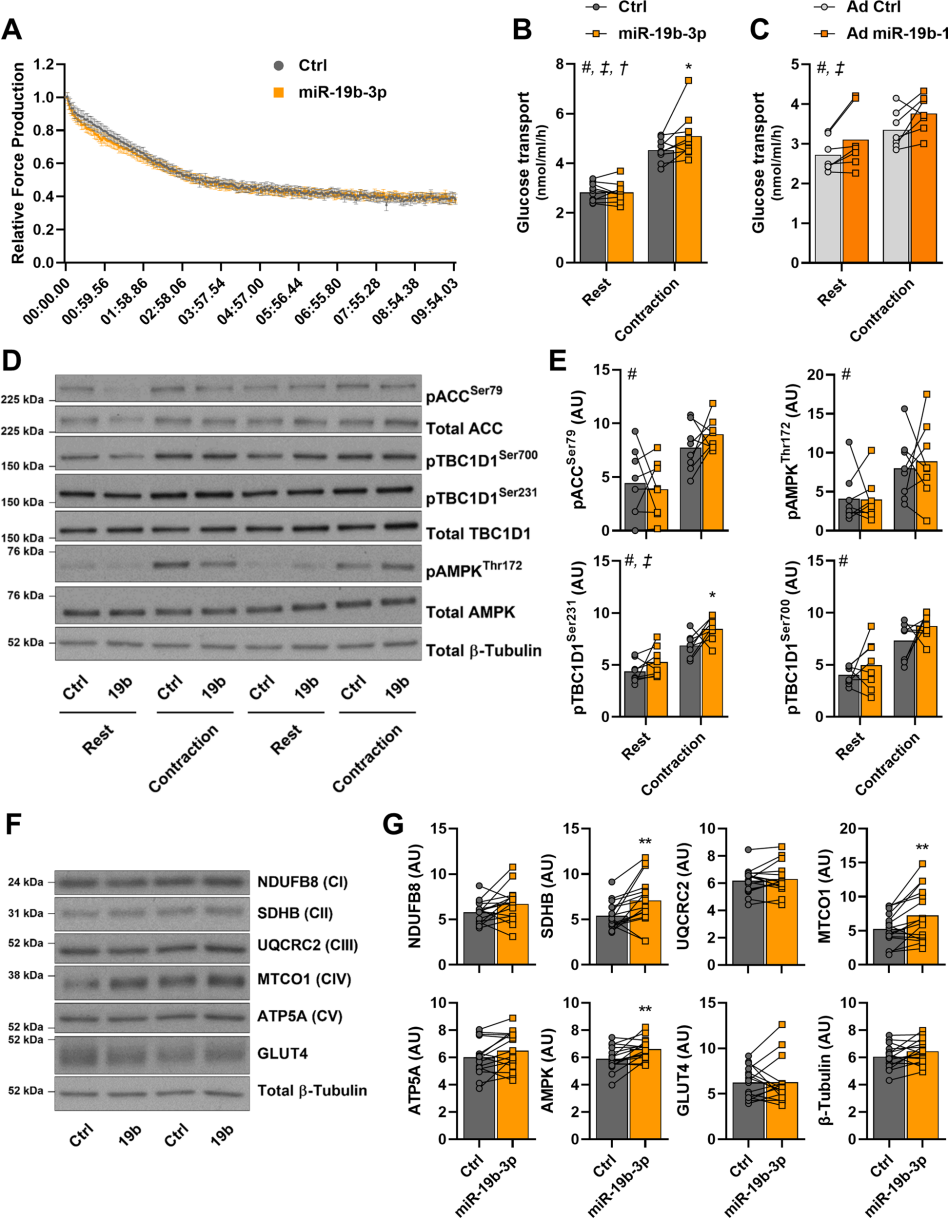
miR-19b-3p improves contraction-stimulated glucose transport in mouse skeletal muscle. Mouse flexor digitorum brevis (FDB) skeletal muscle was electroporated with a control (Ctrl) or a pri-miR-19b-3p-encoding plasmid (miR-19b-3p) and 1 week later, was subjected to electrical pulse-stimulated contraction in vitro. **A** Relative force produced during each contraction measured over 10 min (*n* = 9 independent mouse muscles per construct). **B** In vitro glucose transport in FDB skeletal muscle electroporated with either control or pri-miR-19b-3p-encoding plasmid at rest or following electrical pulse-stimulated contraction (*p* = 0.0163) (*n* = 10 for rest and *n* = 9 for contraction independent mouse muscles per construct). **C** In vitro glucose transport in FDB skeletal muscle transduced with a control (Ad Ctrl) or *mmu-miR-19b-1*-expressing adenovirus (Ad miR-19b-1) at rest or following electrical pulse-stimulated contraction (*n* = 7 independent mouse muscles). **D** Representative immunoblots of FDB skeletal muscle to determine the effects of miR-19b-3p overexpression on basal and contraction-induced protein phosphorylation or abundance. **E** Quantification of (**D**) for phosphorylation of ACC (Ser^79^), AMPK (Thr^172^), and TBC1D1 (Ser^231^ and Ser^700^) (pTBC1D1 Ser^231^: *p* = 0.0479 following contraction) (*n* = 8 independent mouse muscles). **F** Representative immunoblots of FDB skeletal muscle to determine the effects of miR-19b-3p overexpression on protein abundance. **G** Quantification of (**F**) for total abundance of NDUFB8, SDHB (*p* = 0.0085), UQCRC2, MTCO1 (*p* = 0.005), ATP5A, AMPK (α-subunits; *p* = 0.0025), and GLUT4 (*n* = 16 independent mouse muscles). Data are mean. # Contraction effect; ‡ miRNA effect; † Interaction; and *p < 0.05 and **p < 0.01 using two-tailed paired Student’s *t*-test or repeated measures two-way ANOVA with Sidak’s post hoc testing when appropriate.

To validate the role of miR-19b-3p on glucose metabolism in vivo, we introduced a pri-miR-19b-3p-encoding plasmid into mouse flexor digitorum brevis (FDB) muscle by electroporation. Seven days after electroporation the expression of miR-19b-3p was increased ~2-fold (Supplementary Fig. 3A). Overexpression of miR-19b-3p did not alter contractile properties in response to electrical pulse-stimulated contraction; relative force production over the time-course of contraction, maximal force production, or time to 50% fatigue were unaltered (Fig. 3A and Supplementary Fig. 3B, C). Interestingly, miR-19b-3p overexpression was associated with an enhanced contraction-induced glucose uptake, whereas basal glucose transport was unaffected (Fig. 3B). We further assessed the effect of miR-19b-3p on glucose uptake in vivo with adenoviral vectors expressing *miR-19b-1* gene. Viral infection resulted in <2-fold overexpression of mature miR-19b-3p as compared to control GFP muscles (Supplementary Fig. 3D). An overall effect of miR-19b-3p overexpression to increase glucose uptake in intact muscle was observed (Fig. 3C). Contraction-induced phosphorylation of TBC1D1 at Ser^231^ (human Ser^237^) was increased following miR-19b-3p overexpression, while Ser^700^ phosphorylation was unaltered (Fig. 3D, E). TBC1D1 is rapidly phosphorylated at Ser^231^ following skeletal muscle contraction by its upstream kinase AMP-activated protein kinase (AMPK)^14^, leading to increased translocation of GLUT4-containing vesicles and glucose transport. Intriguingly, overexpression of miR-19b-3p did not affect basal or contraction-induced phosphorylation of AMPK or its other downstream target acetyl-CoA carboxylase (ACC) (Fig. 3D, E). Although AMPK phosphorylation was unaffected, total abundance of AMPK α-subunits was increased by miR-19b-3p overexpression (Fig. 3D, E, G). In addition, miR-19b-3p overexpression increased abundance of mitochondrial complex subunits, indicating that oxidative phosphorylation is enhanced since abundance of succinate dehydrogenase complex iron sulfur subunit B (SDHB; complex II subunit) and mitochondrially encoded cytochrome c oxidase I (MTCO1; complex IV subunit) was increased (Fig. 3F, G). Abundance of other mitochondrial complex subunits (NDUFB8, UQCRC2, and ATP5A) and of GLUT4 was unaltered (Fig. 3F, G). Insulin-stimulated (0.36 nM) glucose transport and phosphorylation of Akt and GSK3α was unaltered by miR-19b-3p overexpression (Supplementary Fig. 3E–G). Thus, miR-19b-3p overexpression increases abundance of mitochondrial protein complexes and potentiates contraction-induced glucose uptake in intact skeletal muscle.

### miR-19b-3p modulates expression of genes altered by endurance exercise training in human skeletal muscle

**Fig. 4:**
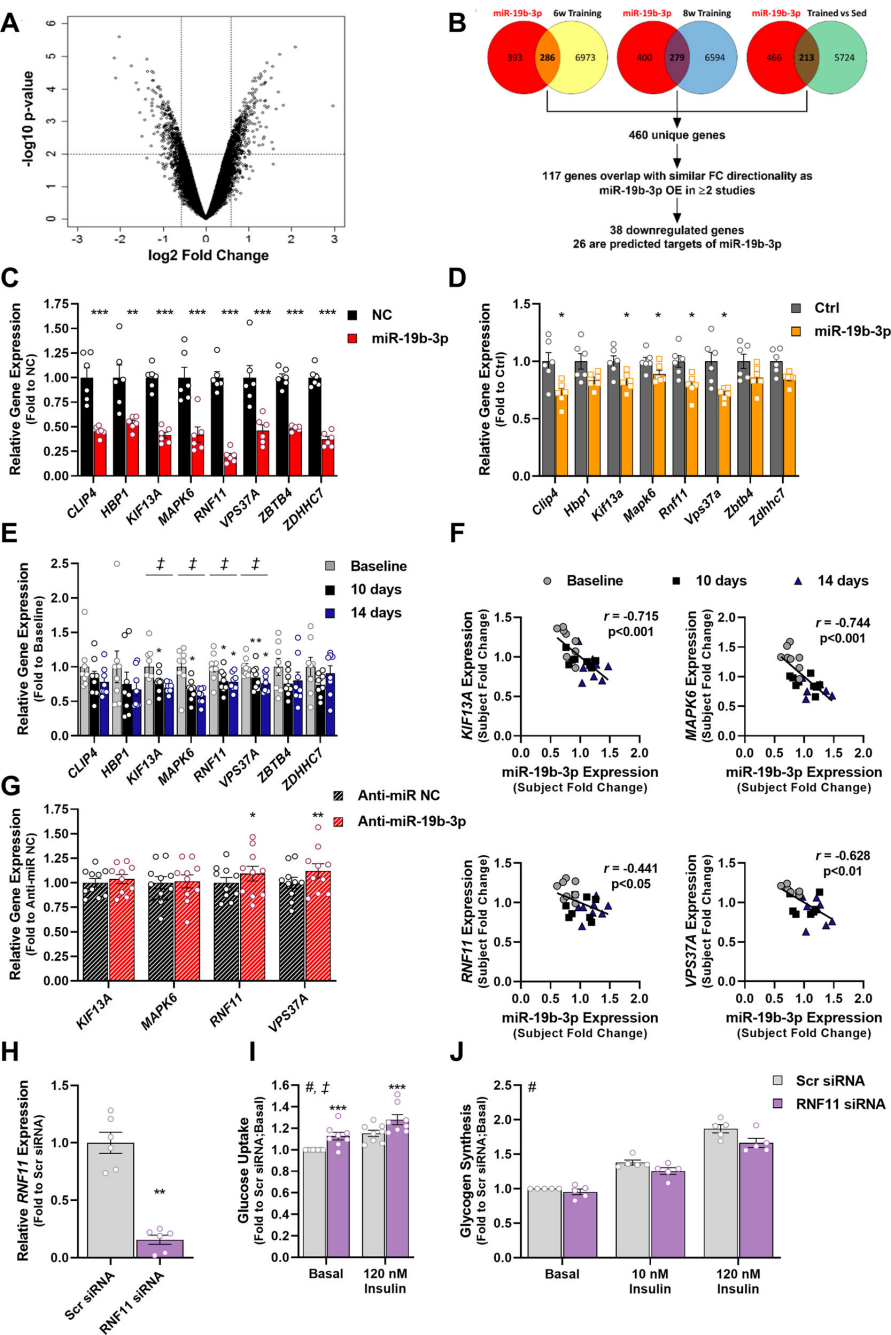
miR-19b-3p modulates expression of genes altered by endurance exercise training in human skeletal muscle. **A** Volcano plot showing changes in gene expression determined by microarray analysis of myotubes overexpressing miR-19b-3p. Dashed lines indicate value cut-off at *p* < 0.01 and fold change of at least ±50% (*n* = 4 independent cell culture experiments). **B** Overview of workflow to identify genes with altered expression profiles following miR-19b-3p overexpression in human skeletal muscle cells and following aerobic exercise training (analysis of publicly available data^8,15,16^). **C** Expression of miR-19b-3p predicted target genes *CLIP4* (*p* = 0.0001), *HBP1* (*p* = 0.0032), *KIF13A* (*p* = 0.0001), *MAPK6* (*p* = 0.0007), *RNF11* (*p* = 0.0001)*, VPS37A* (*p* = 0.0004)*, ZBTB4* (*p* = 0.0001)*,* and *ZDHHC7* (*p* = 0.0001) as determined by RT-qPCR following negative control (NC) or miR-19b-3p overexpression in human skeletal muscle cells (*n* = 6 independent cell culture experiments). **D** Effect of miR-19b-3p overexpression in mouse tibialis anterior skeletal muscle on expression of *Clip4* (*p* = 0.0275)*, Hbp1*, *Kif13a* (*p* = 0.0388), *Mapk6* (*p* = 0.0116), *Rnf11* (*p* = 0.034)*, Vps37a* (*p* = 0.0206)*, Zbtb4*, and *Zdhhc7* as determined by RT-qPCR (*n* = 6 independent mouse samples). **E** Gene expression of *CLIP4, HBP1, KIF13A* (*p* = 0.0497 at day 10), *MAPK6* (*p* = 0.0143 at day 10 and *p* = 0.0031 at day 14)*, RNF11* (*p* = 0.038 at day 10 and *p* = 0.017 at day 14)*, VPS37A* (*p* = 0.0051 at day 10 and *p* = 0.0222 at day 14)*, ZBTB4*, and *ZDHHC7* as determined by RT-qPCR in human skeletal muscle biopsies collected before (Baseline) and after 10 or 14 days of endurance exercise training (*n* = 8 individuals). **F** Pearson correlation of fold changes in miR-19b-3p expression and fold change of *KIF13A* (*p* = 0.0001)*, MAPK6* (*p* = 0.0001)*, RNF11* (*p* = 0.0312), or *VPS37A* (*p* = 0.001) expression in human skeletal muscle biopsies collected prior to (Baseline) and after 10 or 14 days of endurance exercise training (*n* = 24 biopsies). **G** Human myotubes were transfected with a miR-19b-3p-specific inhibitor (Anti-miR-19b-3p) or a negative control inhibitor (Anti-miR NC) and gene expression of *KIF13A*, *MAPK6*, *RNF11* (*p* = 0.024), and *VPS37A* (*p* = 0.0034) was determined by RT-qPCR (*n* = 10 independent cell culture experiments). **H** Human skeletal muscle myotubes were transfected with a scramble siRNA sequence (Scr siRNA) or a RNF11 siRNA and gene expression of RNF11 was determined by RT-qPCR (*p* = 0018, *n* = 6 independent cell culture experiments). **I** Human skeletal muscle myotubes were incubated in the absence (Basal, *p* = 0.0004) or presence of insulin (120 nM, *p* = 0.0008) to determine the effect of RNF11 silencing on glucose uptake (*n* = 8 independent cell culture experiments). **J** Glycogen synthesis rate was determined in human skeletal muscle cells in the absence (Basal) or presence of insulin (10 or 120 nM) following RNF11 silencing (*n* = 5 independent cell culture experiments). Data are mean ± SEM. RT-qPCR data in human skeletal muscle biopsies and cells were normalized to the geometrical mean of *GUSB* and *TBP* expression. For mouse TA muscle, gene expression was normalized to *Tbp* expression. # Insulin effect; ‡ Endurance exercise training effect; and *p < 0.05, **p < 0.01, ***p < 0.001 by two-tailed paired Student’s *t*-test, repeated measures one-way ANOVA with Dunnett’s post hoc testing, or repeated measures two-way ANOVA with Sidak’s post hoc testing when appropriate.

To identify the miR-19b-3p targets that control glucose metabolism, we first performed transcriptional profiling by microarray analysis of human skeletal muscle cells following miR-19b-3p overexpression. miR-19b-3p altered the expression of 679 genes (Fig. 4A and Supplementary Data 1), of which 208 were predicted targets of miR-19b-3p using the prediction target algorithms TargetScan and microRNA.org. The majority (75%) of the predicted target genes were downregulated. Gene Ontology (GO) enrichment analysis of differentially expressed genes in miR-19b-3p overexpressing cells returned enrichment for genes related to extracellular matrix (ECM) organization (Supplementary Table 3). Since miR-19b-3p abundance was increased after aerobic exercise training, we aimed to discover putative exercise training-regulated genes under the control of these miRNAs in human skeletal muscle. Therefore, we compared the gene expression profile of human skeletal muscle cells following miR-19b-3p overexpression against published transcriptome analysis of human skeletal muscle biopsies obtained before and after aerobic exercise training^8,15,16^. These three exercise training studies compared gene expression of skeletal muscle from sedentary versus well-trained individuals^15^, or from healthy sedentary volunteers undergoing either a 6-week^8^ or an 8-week^16^ aerobic exercise-training program. Using a non-stringent *p*-value cutoff of *p* < 0.05, we identified 460 unique genes altered by overexpression of miR-19b-3p and in at least one of the aerobic exercise-training studies (Fig. 4B). Of those, we identified 26 miR-19b-3p putative target genes overlapping at least two exercise training studies and having a similar fold change directionality following miRNA overexpression and after training (Fig. 4B and Supplementary Table 4). We validated a subset of these predicted miR-19b-3p target genes by RT-qPCR following miR-19b-3p overexpression in human skeletal muscle cells, including *CLIP4*, *HBP1*, *KIF13A*, *MAPK6*, *RNF11*, *VPS37A*, *ZBTB4*, and *ZDHHC7* (Fig. 4C). Similar results, with the exception of *Zbtb4*, were observed in mouse skeletal muscle cells following miR-19b-3p overexpression (Supplementary Fig. 4A). Overexpression of miR-19b-3p in mouse tibialis anterior (TA) muscle reduced the expression of *Clip4*, *Kif13a*, *Mapk6*, *Rnf11*, and *Vps37a* (Fig. 4D and Supplementary Fig. 4B). Seven days post-transduction, adenovirus-mediated mature miR-19b-3p overexpression in FDB muscle reduced the expression of 6 of the selected target genes, namely *Clip4, Hbp1, Kif13a*, *Mapk6*, *Rnf11*, and *Vps37a* (Supplementary Fig. 4C). Finally, we determined the expression of these eight miR-19b-3p predicted target genes in skeletal muscle biopsies of individuals undergoing aerobic exercise training. We found that aerobic exercise training reduced the expression of *KIF13A*, *MAPK6*, *RNF11*, and *VPS37A* (Fig. 4E). We found an inverse correlation between the training-induced fold change in miR-19b-3p expression and the expression of each of these four genes (Fig. 4F). Of interest, inhibition of miR-19b-3p in human skeletal muscle cells increased expression of *RNF11* and *VPS37A* (Fig. 4G), further underscoring that miR-19b-3p directly regulates these genes. To determine the effects of miR-19b-3p target genes on glucose metabolism, we silenced *KIF13A*, *MAPK6*, *RNF11*, or *VPS37A* in human skeletal muscle cells (Fig. 4H and Supplementary Fig. 4D). Silencing of RNF11 increased basal and insulin-stimulated glucose uptake, mimicking miR-19b-3p overexpression (Fig. 4I). RNF11 silencing did not alter basal or insulin-stimulated glycogen synthesis (Fig. 4J). Conversely, silencing the other genes did not alter glucose uptake or glycogen synthesis in human skeletal muscle cells (Supplementary Fig. 4E–J). Thus, while RNF11 may contribute to the miR-19b-3p-induced alterations of glucose uptake, it alone is unlikely to explain the full spectra of metabolic changes induced by miR-19b-3p.

## Discussion

miRNAs are post-transcriptional regulators involved in fine-tuning several biological processes. Using an unbiased short RNA sequencing approach, we describe the miRNA profile of human skeletal muscle and identify aerobic exercise-responsive miRNAs in healthy young volunteers. We show aerobic exercise training alters the expression of several miRNAs in skeletal muscle, including miR-19b-3p. Overexpression of miR-19b-3p in cultured human skeletal muscle cells increases glucose uptake and oxidation. Furthermore, miR-19b-3p overexpression increases canonical insulin signaling at the level of Akt and AS160, consistent with enhanced insulin sensitivity. Furthermore, our efforts to mimic the profile of exercise-trained muscle through overexpression of miR-19b-3p in intact mouse skeletal muscle, provided evidence that miR-19b-3p controls contraction-mediated glucose uptake. Transcriptomic analysis revealed networks regulated by miR-19b-3p involved in skeletal muscle remodeling and metabolism. miR-19b-3p regulates several targets found to be altered in response to aerobic exercise training, including *KIF13A*, *MAPK6*, or *RNF11*. Silencing of RNF11, a protein previously unstudied in skeletal muscle, resulted in an increased glucose uptake. Thus, RNF11 may partially account for exercise- and miR-19b-3p-mediated increase in skeletal muscle glucose metabolism.

Exercise training-responsive miRNAs have been explored by both unbiased microarray and RT-qPCR analysis of selected candidate miRNAs^8,10^. These approaches depend on prior sequence knowledge. In contrast, RNA sequencing requires no prior sequence knowledge of constituent RNA. The RNA sequencing data reported in this study provides an unbiased miRNA expression profile of untrained and aerobic exercise-trained human skeletal muscle. Consistent with previous studies^8,10^, we found that aerobic exercise training reduced the expression of several members of the myomiR family, including miR-133a-5p, miR-1-5p, and miR-133b. Training-specific responses on myomiR expression have been reported. Aerobic exercise training reduced myomiR expression, while acute aerobic exercise increased miR-1 and miR-133 expression^10^. In contrast, resistance exercise training had no effect^17^. The training-specific alterations of myomiR expression may arise from remodeling processes that depend on intensity, duration, and type of exercise.

Skeletal muscle is a complex tissue, and the effects of exercise-training are not solely restricted to myocytes. We identified four miRNAs that were increased in skeletal muscle after exercise training; miR-19b-3p, miR-107, miR-223-3p, and miR-451a. miR-19b-3p and miR-107 are expressed in myocytes, whereas miR-223-3p and miR-451a are likely expressed in cells of non-muscle origin. miR-451a is enriched in erythrocytes, with relatively low abundance in skeletal muscle^18^, and circulating levels of miR-223-3p are increased following endurance exercise, with expression levels linked to the inflammatory response associated with exercise^19,20^. Besides acting intracellularly, miRNAs can be secreted to neighboring cells or into the circulation, and therefore they may be involved in cell-to-cell signaling and communication^21^. Thus, miR-223-3p and miR-451a may act in a paracrine manner in skeletal muscle in response to exercise training. Skeletal muscle expression of miR-107 is increased following acute treadmill running^22^ and decreased by hind-limb suspension^23^ in mouse models. We report that miR-107 alters differentiation of human myotubes, consistent with findings of reduced myogenesis in bovine skeletal muscle cells^24^. Thus, miR-107 appears to play a role in skeletal muscle remodeling. Given that the differentiation state has a profound effect on metabolism^13^, we deprioritized further studies on miR-107. Consequently, we selected the exercise-regulated miR-19b-3p for further validation to gain insight into mechanisms by which aerobic exercise training alters insulin sensitivity and metabolism in skeletal muscle.

The molecular networks contributing to the adaptation to exercise in human skeletal muscle are not fully identified. While specific transcription factors and co-factors, such as NR4As, PPARs, or PGC-1α are involved in the response to a single bout of exercise, there is a coordinated transcriptional response to exercise which is both time- and intensity-dependent^7^. Some genes are regulated during and directly after exercise (for example TFAM)^25^ and others are altered in the recovery (~3 h) and adaptive (>12 h) phases following exercise (such as PGC1α and PPARδ)^25^. We provide evidence that miR-19b-3p expression is induced 19 h post-exercise cessation and that elevation of miR-19b-3p transcripts requires moderate- to high-intensity exertion. Since other members of the miR-17/92 cluster were also increased, it is likely that elevated miR-19b-3p levels after exercise training is driven by transcriptional regulation, and not regulation of miR-19b-3p stability. Myc is a candidate transcription factor regulating miR-19b-3p transcription as it is induced within 30 min post-exercise^26^ and can directly bind to the promoter of the miR-17/92 cluster and initiate transcription^27^. However, we cannot exclude that miR-19b-3p is also independently regulated by other post-transcriptional mechanisms, such as acetylated AGO2 favoring the production of mature miR-19b-3p from the pre-miRNA^28^. Overall, we show that miR-19b-3p expression is increased in the adaptive phase (>12 h) after exercise training and may play a role in directing muscle remodeling following moderate to high-intensity exercise. The increase in miR-19b-3p is maintained during 14 days of endurance training.

Regular exercise improves insulin sensitivity, glucose metabolism, and mitochondrial biogenesis in skeletal muscle^7^, but the role of miRNAs in orchestrating these changes remains largely unknown. Thus, we assessed whether miR-19b-3p, an exercise training-induced miRNA, affects skeletal muscle metabolism. In cultured human skeletal muscle cells, inhibition of endogenous miR-19b-3p reduced insulin-stimulated glucose uptake, and overexpression of miR-19b-3p enhanced glucose uptake. Likewise, in mouse skeletal muscle, a modest overexpression of miR-19b-3p enhanced contraction-induced glucose transport, similar to exercise training^29^. While the effect size of the increase in miR-19b-3p enhanced glucose uptake was modest, given that skeletal muscle mass represents up to 50% of total body mass, even small enhancements are likely to significantly impact whole-body glucose homeostasis. The duration and magnitude in miR-19b-3p overexpression achieved in mouse muscle were similar to that noted in response to exercise training in the young healthy men, supporting a physiological role of miR-19b-3p in regulation of glucose transport. These improvements in glucose uptake may be linked to changes in signal transduction. We found TBC1D1^Ser231^ phosphorylation was increased by miR-19b-3p overexpression, consistent with the response of this signal transducer to acute exercise^30^. We further determined the metabolic fate of glucose in response to miR-19b-3p overexpression in myotubes, and noted increased oxidation and incorporation into glycogen. Increased glucose transport and glycogen synthesis are hallmarks of exercise training^31^, however, increased lipid oxidation is another mechanism by which exercise training improves metabolism^32^. We found miR-19b-3p overexpressing cells preferentially oxidized glucose over lipids, which may reflect the predominantly glycolytic cell culture conditions, rather than the substrates available to the working muscle during exercise. In intact mouse muscle, miR-19b-3p overexpression increased the abundance of mitochondrial proteins, consistent with effects of exercise training on the mitochondrial proteome^33^. Overall our results provide evidence that miR-19b-3p recapitulates part of the effects of exercise training on mitochondrial markers and glucose metabolism.

The identification of target genes mediating the functional effects of miRNAs is challenging. We applied a transcriptomic approach to determine the effects of miR-19b-3p overexpression on gene expression in human skeletal muscle cells. We found that miR-19b-3p overexpression in myotubes regulates genes involved in extracellular matrix (ECM) remodeling, a process implicated in exercise-training-related adaptations in skeletal muscle, with expression of ECM genes linked to the magnitude of the aerobic exercise training response^34^. This suggests that in addition to the regulation of glucose metabolism, miR-19b-3p may also control processes involved in maintaining the structural integrity and functional properties of skeletal muscle^35^. However, we did not observe marked change of force production in intact mouse muscle following a modest overexpression of miR-19b-3p. Of note, flexor digitorum brevis muscle is more fatigue-resistant than other muscle types^36^, thus we cannot exclude the possibility that the electrical stimulation protocol (strength versus endurance) or type of muscle studied (fatigue prone versus fatigue resistant) influenced these results.

To ascertain whether the supraphysiological overexpression of miR-19b-3p drives non-specific effects, including alterations in gene expression measured by microarray, we compared the myotube transcriptomic profile with that of endurance-trained skeletal muscle^8,15,16^. We identified 26 predicted targets downregulated both by miR-19b-3p overexpression and aerobic exercise training. We confirmed that several of these predicted targets, including *KIF13A*, *MAPK6*, *RNF11*, and *VPS37A*, were also regulated by exercise training in our cohort. RNF11 silencing enhanced basal and insulin-stimulated glucose uptake, similar to miR-19b-3p overexpression. RNF11 is a miR-19b-3p validated target by luciferase assay^37^, and is an essential component of ubiquitin-editing, implicated in NF-κB activation and inflammation^38^. RNF11 has a 14-3-3 binding sequence and an Akt phosphorylation site^39^, potentially linking it to functional events downstream of Akt including growth and glucose metabolism. Thus, the effects of miR-19b-3p on metabolism may be partially mediated by RNF11.

Using short RNA sequencing we describe the human skeletal muscle miRNA profile and identify aerobic exercise training-responsive miRNAs. We validate miR-19b-3p as an aerobic exercise training-induced miRNA that regulates skeletal muscle insulin sensitivity and glucose metabolism. Of the predicted miR-19b-3p targets altered in skeletal muscle after exercise training, RNF11 regulates glucose uptake. In conclusion, miR-19b-3p contributes to exercise training-induced improvements in skeletal muscle partly via RNF11.

## Methods

### Exercise study cohorts

All participants provided written informed consent and the research ethical committee at Dublin City University approved the study protocols and experimental procedures were conducted according to the Declaration of Helsinki. Two separate cohorts were used.

#### Human participants for exercise training study

Eight healthy, sedentary men (23 ± 2 years of age) volunteered to participate in a 14-day consecutive training program as described^33^. The human participants exercised on a cycle ergometer for 1 h at ~80% peak oxygen uptake during each session as described^7^. Skeletal muscle biopsies were taken after an overnight fast prior to the first training session (*n* = 5), and after 10 days, and 14 days (*n* = 8) of exercise training (all biopsies were taken ~16 h after cessation of the last exercise bout). Biopsies were taken from vastus lateralis under local anesthesia using a biopsy needle, snap frozen in liquid nitrogen, and stored at −80 °C.

#### Human participants for acute exercise study

Healthy sedentary male volunteers completed two isocaloric acute exercise bouts at low (40% VO_2peak_) and high (80% VO_2peak_) intensity on two occasions separated by at least 1 week in random order^40^. Skeletal muscle biopsies were obtained at baseline, 3 h, and 19 h after each respective exercise session. A subset of biopsies obtained from five subjects was retrieved for RNA extraction in this study.

### Cell cultures

#### Primary human skeletal muscle cells

Human satellite cells were isolated from vastus lateralis skeletal muscle biopsies of healthy volunteers as described^41^. The research ethical committee at Karolinska Institutet approved the study protocols and all volunteers provided informed consent. Experimental procedures were conducted according to the Declaration of Helsinki. Myoblasts were grown and differentiated into multinucleated myotubes as described^42^. Cells were propagated in growth media (F12/DMEM, 20% FBS, and 1% anti-anti) and when ~90% confluent, cells were differentiated for 4 days with fusion media (DMEM/M199 (4:1), 1% FBS, 1% penicillin-streptomycin/amphotericin B (anti-anti), 20 mM HEPES, 0.03 μg mL^−1^ ZnSO_4_, 1.4 μg mL^−1^ vitamin B12, 10 μg mL^−1^ insulin, and 100 μg mL^−1^ apo-transferrin). Thereafter, cells were cultured in post-fusion media (DMEM/M199 (4:1), 1% FBS, 1% anti-anti, 20 mM HEPES, 0.03 μg mL^−1^ ZnSO_4_, and 1.4 μg mL^−1^ vitamin B12). Cultures were incubated at 37 °C in 7.5% CO_2_ humidified chambers, and medium was changed every second day during growth and differentiation.

Transfection of human skeletal muscle cells with either Pre-miR™ miRNA Precursors (25 nM), mirVana® miRNA inhibitors (25 nM), or Silencer Select siRNAs (5 nM) was performed 6 days after induction of differentiation. Transfections were performed for 5 h in OptiMEM reduced serum media with Lipofectamine® RNAiMAX. A second transfection was conducted after 48 h and all cellular assays were performed 2 days after the second transfection. Media, miRNA precursors and inhibitors, siRNAs, and transfection reagents were from Thermo Fisher Scientific and oligonucleotides used are reported in Supplementary Table 5.

#### Primary mouse skeletal muscle cells

Primary mouse skeletal muscle cells were isolated as described^43^. Hind limb muscles from 2-week-old C57BL/6J mice were removed and mechanically minced in PBS. Thereafter, enzymatic dissociation of satellite cells was performed with Collagenase and Dispase (Thermo Fisher Scientific, MA) and the suspension was passed through a 70 μm cell strainer. Differential plating was performed during the initial passages to enrich for myoblasts. Cells were grown on collagen-coated plates with growth media (DMEM/Ham’s F10 (1:1), 20% FBS, 1% penicillin-streptomycin/amphotericin B (anti-anti), and 2.5 ng mL^−1^ bFGF). Differentiation was induced after plating 83,300 cells cm^−2^ and ~12 h later switching to differentiation media (DMEM, 5% horse serum, and 1% anti-anti). Cultures were incubated at 37 °C in 5% CO_2_ humidified chambers, and medium was changed every second day during growth and differentiation.

Cells were differentiated for 2 days and transfected with miRNA Pre-miR™ miRNA Precursors (25 nM) for 5 h in OptiMEM reduced serum media with Lipofectamine® RNAiMAX. Two days after a single transfection, final experiments were performed. Media, miRNA precursors, and transfection reagents were from Thermo Fisher Scientific.

#### C2C12 skeletal muscle cells

Mouse C2C12 myoblasts (ATCC, VA) were propagated in growth media (DMEM, 20% FBS, and 1% anti-anti). Cells were seeded (42,000 cells cm^−2^) and ~12 h thereafter the culture medium was changed to differentiation medium (DMEM, 2% horse serum, and 1% anti-anti). Cells were incubated at 37 °C in 5% CO_2_ humidified chambers, and medium was changed every second day during growth and differentiation. Cells were differentiated for 3 days and then transfected using a double transfection protocol. Cells were transfected with miRNA Pre-miR™ miRNA Precursors (25 nM) for 5 h in OptiMEM reduced serum media with Lipofectamine® RNAiMAX. For the double transfection protocol, cells were transfected again 48 h after the first transfection and final experiments were performed 2 days later.

### Short RNA sequencing and analysis

Short RNA sequencing of human skeletal muscle was performed using a previously described protocol^44^. Total RNA was extracted from human skeletal muscle biopsies using TRIzol. Small RNA molecules 18–24 nucleotides long were purified using mirVana™ miRNA Isolation Kit (Thermo Fisher Scientific) followed by 15% PAGE/7 M urea. Thereafter, ligation of 3′- and 5′-adaptors was performed, cDNA was synthetized and amplified, and miRNA abundance was determined by sequencing (Illumina, CA). Short RNA sequencing reads were trimmed using FastX followed by mapping to the hg38 reference genome using Bowtie2^45^. miRNA coverages were computed using featureCounts^46^ with miRBase v21 annotations. miRNAs with expression <100 reads per million (RPM) in more than three samples were excluded from the differential expression analysis since they are unlikely to be active^12^. Differential expression of miRNAs was determined using the edgeR glmQLFit/glmQLFTest framework^47^ and corrected for multiple testing using the Benjamini–Hochberg correction.

### Glucose uptake measurements in cellular models

Glucose uptake was determined in primary human skeletal muscle cells as described^42^. Cells were starved of serum for 4 h prior to treatment with insulin. Glucose uptake was determined following incubation of cells for 1 h in absence or presence of insulin, prior to addition of 2-[1,2-^3^H]deoxy-D-glucose (Moravek Inc., CA) and 10 mM unlabeled 2-deoxy-D-glucose for 15 min. Cells were homogenized with 0.03% SDS and the amount of 2-[1,2-^3^H]deoxy-D-glucose in the homogenates was determined by scintillation counting.

Glucose uptake in primary mouse skeletal muscle cells was determined as described^48^. Cells were serum-starved for 4 h before incubation in the absence or presence of insulin for 20 min. Thereafter, cells were rinsed twice with HEPES-buffered saline (140 mM NaCl, 20 mM Na-HEPES, 2.5 mM MgSO_4_, 1 mM CaCl_2_, 5 mM KCl, pH 7.4) and incubated for 5 min (with 2-[1,2-^3^H]deoxy-D-glucose and 10 mM unlabeled 2-deoxy-D-glucose.

C2C12 myotubes were serum-starved for 4 h and then incubated in the absence or presence of insulin. Thereafter, glucose uptake was determined for 15 min in cells incubated with 2-[1,2-^3^H]deoxy-D-glucose and 10 mM unlabeled 2-deoxy-D-glucose. Cells were homogenized with 0.03% SDS and the amount of 2-[1,2-^3^H]deoxy-D-glucose in homogenates was determined by scintillation counting.

Normalization of glucose uptake was performed to total protein content (Pierce BCA Protein Assay Kit, Thermo Fisher Scientific) and scintillation counting was determined using a 1414 WinSpectral Liquid Scintillation Counter (Wallac, Perkin-Elmer, MA). All radioisotopes were acquired from Perkin-Elmer if not otherwise stated.

### Glycogen synthesis analysis in primary human and mouse skeletal muscle cells

Glucose incorporation into glycogen was determined in primary human and mouse skeletal muscle cells as described^42^. Cells were starved of serum for 4 h prior to treatment with insulin and thereafter incubated for 30 min in the absence or presence of insulin followed by addition of D-[U-^14^C]glucose (Perkin-Elmer) for 90 min. Cells were homogenized, glycogen was precipitated with ethanol at −20 °C overnight, and the amount of D-[U-^14^C]glucose-containing glycogen was determined by scintillation counting. Normalization of glucose incorporation into glycogen was performed to total protein content (Pierce BCA Protein Assay Kit, Thermo Fisher Scientific) and scintillation counting was determined using a 1414 WinSpectral Liquid Scintillation Counter.

### Glucose and palmitate oxidation in cells

Glucose and palmitate oxidation were determined in primary human skeletal muscle cells as described^42^. Glucose oxidation was determined by incubation of cells with D-[U-^14^C]glucose in the absence or presence of 1 µM FCCP (Sigma-Aldrich). Plates were sealed and incubated for 4 h. Thereafter, media was acidified (1:8 vol 2 M HCl) and the liberated ^14^CO_2_ was collected for 1 h in a center well containing 2 M NaOH. The amount of liberated ^14^CO_2_ was determined by scintillation counting. Palmitate oxidation was determined following pre-treatment of cells with or without 10 nM GW1516 (GW501516, Sigma-Aldrich) for 96 h. Cells were incubated with 25 µM non-labeled palmitic acid and [9,10-^3^H]palmitic acid for 6 h. The amount of ^3^H_2_O released into culture media was determined by scintillation counting. Results were normalized to total cellular protein content (Pierce BCA Protein Assay Kit, Thermo Fisher Scientific) and scintillation counting was determined using a 1414 WinSpectral Liquid Scintillation Counter.

### Extracellular flux analysis (Seahorse)

Mitochondrial function was assessed using Seahorse XF Cell Mito Stress Test Kit (Agilent Technologies, CA). Cells were plated at a density of 30,000 cells per well in Seahorse XF24 Cell Culture Microplates. Differentiation was initiated the following day with addition of fusion media for 4 days and cells were subsequently cultured for 2 days in post fusion media. A double transfection was performed and the assay was performed 48 h after the second transfection. Cells were incubated in XF Base Medium supplemented with 5 mM glucose, 1 mM pyruvate, and 2 mM L-glutamine. OCR values were obtained at baseline and after addition of 1 μM oligomycin, 2 µM FCCP, and 0.75 μM rotenone + 0.75 μM antimycin A. OCR values were normalized to protein content (Pierce BCA Protein Assay Kit, Thermo Fisher Scientific).

### Animal experiments

All animal experiments were approved by the Regional Animal Ethical Committee (Stockholm, Sweden) and all work was performed in adherence with relevant guidelines. Fifteen-week-old male C57BL/6J mice were purchased from Charles River Laboratories (Sulzfeld, Germany). Animals were housed under a 12-h light/12-h dark cycle, 24 °C and 45% humidity, and received ad libitum access to water and standard rodent chow (Lantmännen, Sweden). Mice were acclimatized for at least 1 week prior to experimental interventions.

#### Transcardial perfusion

Transcardial perfusion with PBS was performed in mice anesthetized with Avertin (2,2,2-tribromoethanol and tertiary amyl alcohol) until the perfused heart was free of blood. Soleus and extensor digitorum longus (EDL) skeletal muscles were removed and immediately frozen in liquid nitrogen for subsequent analysis.

#### Electroporation of mouse skeletal muscle

C57Bl/6J male mice were anesthetized with isoflurane and tibialis anterior (TA) muscles injected with hyaluronidase (30 µl; 1 unit mL^−1^). Mice received carprofen (5 mg kg^−1^) subcutaneously. Mice were then single-housed for 2 h prior to a second anesthesia with isoflurane. Intramuscular injection with 30 µg of a plasmid encoding either pri-miR-19b-3p or a control plasmid (Origene, MD) in the contralateral leg and electroporation was performed by delivering 220 V cm^−1^ as 8 pulses of 20 ms using an electroporator (ECM 830 Electroporator, BTX, Harvard Bioscience company)^49^. One week after electroporation, mice were fasted for 4 h and anesthetized with Avertin. Tibialis anterior muscles were removed and instantly frozen in liquid nitrogen for subsequent analysis. Flexor digitorum brevis (FDB) muscles were electroporated with 20 µg of a plasmid encoding either pri-miR-19b-3p or a control plasmid on the contralateral foot at 100 V cm^−1^ as 20 pulses of 20 ms using an electroporator^50^. One week after electroporation FDB muscles were carefully dissected out and glucose uptake was assessed following contraction.

#### Intramuscular delivery of adenovirus into mouse skeletal muscle

Human Type 5 (dE1/E3) adenoviruses expressing green fluorescent protein (GFP) alone or *mmu-miR-19b1* and GFP under independent CMV promoters (Vector Biolabs, Malvern, PA) were injected into FDB muscles of 3-week-old C57Bl6J (2 μL/g of mice, 2 × 1010 infectious particles)^51^. Seven days post-injection FDB muscles were carefully dissected out and glucose uptake was assessed following contraction.

### Glucose uptake in mouse flexor digitorum brevis skeletal muscle in vitro

Glucose uptake in electroporated FDB skeletal muscle was assessed following contraction or insulin-stimulation, as previously described^52^. Incubations were performed at 30 °C in Krebs-Henseleit bicarbonate (KHB) buffer supplemented with 0.1% BSA (RIA grade) that were continuously gassed with 95% O_2_ and 5% CO_2_. Insulin-stimulated glucose uptake was determined following a recovery period of 30 min and thereafter FDB muscles were incubated in the absence or presence of insulin (0.36 nM) for 30 min in KHB containing 5 mM glucose and 15 mM mannitol. Contraction-induced glucose uptake was performed using a myograph system (DMT A/S, Denmark) and recorded and analyzed using Labchart v7 software (ADInstruments, New Zealand). Paired FDB muscles were placed in KHB containing 5 mM glucose and 15 mM mannitol for a 20-min-period before being subjected to either electrical stimulation (100 Hz, 0.2 ms pulse duration, ~20 V, one contraction of 0.2 s every 2 s) or rest condition for 10 min. After contraction or insulin-stimulation, muscles were incubated in KHB supplemented with 20 mM mannitol for 10 min before incubation in KHB supplemented with 1 mM 2-deoxyglucose and [^3^H]2-deoxy-glucose (2.5 μCi mL^−1^, American Radiolabeled Chemicals, MO) and 19 mM mannitol and [^14^C]mannitol (0.7 mCi mL^−1^, Moravek Inc., CA) for 20 min. Thereafter, muscles were stripped of connective tissue and snap frozen in liquid nitrogen and later homogenized in homogenization buffer (137 mM NaCl, 2.7 mM KCl, 1 mM MgCl_2_, 0.5 mM Na_3_VO_4_, 1% (vol/vol) Triton X-100, 10% (vol/vol) glycerol, 20 mM Tris, 10 mM NaF, 1 mM EDTA, 1 mM PMSF, and 1% (vol/vol) Protease Inhibitor Cocktail Set 1 (Merck Millipore, MA)). Glucose uptake was determined by scintillation counting and was normalized to protein content (Pierce BCA Protein Assay Kit, Thermo Fisher Scientific).

### RNA isolation and miRNA and gene expression analysis by real-time qPCR

Total RNA from human skeletal muscle biopsies, mouse skeletal muscles and cells were extracted using TRIzol according to manufacturer’s instructions (Thermo Fisher Scientific). RNA concentration was determined by spectrophotometry using a Nanodrop ND-1000 (Thermo Fisher Scientific) and equal amounts of RNA were used for cDNA synthesis. cDNA was synthesized using the High Capacity cDNA Reverse Transcription kit with random primers or the TaqMan™ Advanced miRNA cDNA Synthesis kit (Thermo Fisher Scientific). RT-qPCR was performed using either a ViiA 7 Real-Time PCR 384-well system or a StepOne Plus Real-Time PCR 96-well system (Thermo Fisher Scientific). For determination of relative miRNA abundance, TaqMan Advanced miRNA primers (Supplementary Table 5) and TaqMan® Fast Advanced Master Mix were used according to manufacturer’s recommendation (Thermo Fisher Scientific) and normalization was performed to miR-186-5p expression (Thermo Fisher Scientific recommended reference miRNA). Gene expression was determined using Fast SYBR™ Green Master Mix (Thermo Fisher Scientific) and primers used are reported in Supplementary Table 6. Gene expression in human skeletal muscle biopsies and cells was normalized to the geometrical mean of *GUSB* and *TBP* expression. For C2C12 cells, primary mouse skeletal muscle cells and mouse TA muscle, gene expression was normalized to *Tbp* expression.

### Transcriptomic analysis and identification of miR-19b-3p targets

RNA from human skeletal muscle cells transfected with Pre-miR™ miRNA Precursors for miR-19b-3p or Pre-miR™ miRNA Negative Control was subjected to a transcriptomic analysis. Total RNA (100 ng) was used to generate amplified sense strand DNA followed by fragmentation and biotinylation using the WT Plus Reagent kit (Thermo Fisher Scientific). ssDNA was hybridized to Clariom S gene array chips (Affymetrix, CA). Array chips were washed, stained, and scanned using a Gene Titan instrument (Thermo Fisher Scientific).

Preprocessing of data was performed using Robust Multiarray Average (RMA) using the oligo package^53^ with the R software^54^. After removal of non-annotated genes, the probe level expression was collapsed to gene level (average method) using the WGCNA package^55^. Differentially expressed genes (DEGs) were determined with the linear models for microarray (limma) package^55^, following overexpression of either miR-19b-3p relative to Negative Control transfected cells. DEGs were determined by a paired test and defined as *p*-value < 0.01 and at least 1.5-fold change. Gene ontology (GO) enrichment analysis was performed with DEGs as input using clusterProfiler^56^. The transcriptomic data are available through NCBI’s GEO with accession number GSE126187.

To find miR-19b-3p predicted target genes altered by aerobic exercise training, three publically available microarray studies were utilized. Data from GSE35661 compares vastus lateralis biopsies from 24 healthy sedentary male volunteers before and after 6 weeks of aerobic exercise training^8^, data from GSE27536 compares vastus lateralis biopsies from 12 healthy volunteers (10 males and 2 females; patients with chronic obstructive pulmonary disease were excluded from analysis) before and after a 8-week aerobic exercise program^16^, and data from GSE9103 compares vastus lateralis biopsies from young volunteers who were either sedentary (5 women, 6 men) or exercise-trained (5 women, 6 men; self-reported exercise of at least 1 h of cycling or running 6 days per week over the past 4 years)^15^. Data was generated from NCBI’s GEO depository and analyzed with either GEO2R (GSE9103) or in-house using R with RMA preprocessing (GSE27536 and GSE35661 using paired statistical analysis). Potential aerobic exercise-responsive miR-19b-3p-regulated genes were defined as having (1) altered expression in at least two exercise training studies (*p* < 0.05), (2) similar fold change directionality comparing miRNA overexpression and following exercise training, and (3) being predicted as miR-19b-3p targets using either TargetScan (release 7.1)^57^ or microRNA.org^58^.

### Protein extraction and western blot analysis

Western blot was utilized for determination of protein abundance and phosphorylation status in skeletal muscle cells and tissue. FDB muscles were frozen immediately following glucose transport experiments. Human skeletal muscle cells were serum-starved for 4 h and thereafter incubated in the absence or presence of insulin for 10 min. Cells and tissues were lysed in ice-cold homogenization buffer (137 mM NaCl, 2.7 mM KCl, 1 mM MgCl_2_, 0.5 mM Na_3_VO_4_, 1% (vol/vol) Triton X-100, 10% (vol/vol) glycerol, 20 mM Tris, 10 mM NaF, 1 mM EDTA, 1 mM PMSF, and 1% (vol/vol) Protease Inhibitor Cocktail Set 1 (Merck Millipore, MA)). Lysates were rotated at 4 °C for 30 min before being subjected to centrifugation at 12,000 × *g* at 4 °C for 15 min. Protein concentration was determined by Pierce BCA Protein Assay Kit (Thermo Fisher Scientific) and equal amounts of protein were diluted with Laemmli buffer. Samples were heated at 56 °C for 15 min before being separated by SDS-PAGE using Criterion XT Bis-Tris Gels (Bio-Rad, CA). Proteins were transferred to PVDF membranes (Merck Millipore) and Ponceau S staining was used to confirm equal loading and transfer (Sigma-Aldrich, MO). Membranes were blocked in 7.5% milk in TBST (10 mM Tris-HCl, 100 mM NaCl, 0.02% Tween 20) for 1 h at RT and thereafter incubated at 4 °C overnight with primary antibodies (listed in Supplementary Table 7). Membranes were washed with TBST, incubated for 1 h at RT with appropriate secondary antibodies, washed with TBST, and then visualized by enhanced chemiluminescence (ECL Western Blotting Detection Reagent, GE Healthcare, IL). Protein content was quantified by densitometry (QuantityOne, Bio-Rad). Full scan blots are presented in the Source data file.

### Quantification and statistical analysis

Data are presented as mean ± SEM (if not otherwise stated in Figure Legends). Statistical analysis of RNA sequencing and gene array analysis are described above. Normal distribution of the data was tested with Shapiro–Wilk test. Differences in miRNA and mRNA expression in human biopsies were determined by paired Student’s *t*-test or repeated-measures ANOVA followed by Dunnett’s test for post hoc pair-wise comparisons or using Friedman’s test followed by Dunn’s multiple comparison test when appropriate. For cell culture and animal experiments, miRNA and gene expression were analyzed by paired Student’s *t*-test and metabolic assays were analyzed using repeated-measures or two-way ANOVA followed by appropriate test for post hoc pair-wise comparisons. Time to 50% fatigue in FDB skeletal muscle was determined by plotting a logarithmic line to data representing force production over time during the contraction time-course. Pearson correlations between exercise training-induced fold change in miRNA and gene expression was performed following centring of each individuals genes and miRNAs expression to the individuals average expression (defined as FoldExpression_Timepoint:n_ = Expression_Timepoint:n_/average (Expression_timepoint:Baseline_ + Expression_timepoint:10days_ + Expression_timepoint:14days_)) to reduce variation between subject’s baseline expression to study exercise training-specific effects on gene and miRNA expression. Comparisons were considered statistically significant at *p* < 0.05 and analyses were performed using GraphPad Prism 8 software (GraphPad Software Inc., CA).

### Reporting summary

Further information on research design is available in the Nature Research Reporting Summary linked to this article.

## Supplementary information

Supplementary Information

Description of Additional Supplementary Information

Supplementary Data 1

Reporting summary

## Data Availability

Short RNA-seq data generated in this study have been deposited in the NCBI’s GEO database under accession number GSE127187. The GEO accession number for the gene array-generated transcriptomic data reported in this paper is GSE126187. Raw data of all figures and uncropped versions of blots presented in the figures are provided as a Source data file. The NCBI’s GEO accession numbers for published microarray data analyzed in this paper are GSE35661^8^, GSE27536^16^, and GSE9103^15^. Source data are provided with this paper.

## Source data

Source Data

## Code availability

R scripts for the main steps of analysis are available from the corresponding author on reasonable request.
