# Supplementary material for: Endurance exercise training-responsive miR-19b-3p improves skeletal muscle glucose metabolism
Source: Nat Commun. 2021 Oct 12;12:5948. doi: 10.1038/s41467-021-26095-0 (PMC8511155; doi:10.1038/s41467-021-26095-0)
Supplement: Supplementary file 1 — Supplementary Information [file 41467_2021_26095_MOESM1_ESM.pdf]

Supplemental Information

Endurance Exercise Training-Responsive miR-19b-3p Improves Skeletal Muscle Glucose Metabolism

Julie Massart[1,*], Rasmus J.O. Sjögren[1,*], Brendan Egan[2,3], Christian Garde[4], Magnus Lindgren[1], Weifeng Gu[5,7], Duarte MS Ferreira[6], Mutsumi Katayama[2], Jorge L Ruas[6], Romain Barrès[4], Donal J. O'Gorman[3], Juleen R. Zierath[1,2,4], and Anna Krook[2*]

Running Title: miRNA-dependent regulation of metabolism following exercise training

Affiliations: [1]Department of Molecular Medicine and Surgery, Section of Integrative Physiology, Karolinska Institute, Stockholm, Sweden; [2]Department of Physiology and Pharmacology, Section of Integrative Physiology, Karolinska Institutet, Stockholm, Sweden; [3]Department of Health & Human Performance, Dublin City University, Ireland; [4]Novo Nordisk Foundation Center for Basic Metabolic Research, University of Copenhagen, Denmark; [5] University of Massachusetts Medical School, Worchester, MA Department of Cell Biology and Neuroscience, University of California at Riverside, Riverside, California, USA; and [6]Department of Physiology and Pharmacology, Section of Molecular & Cellular Exercise Physiology, Karolinska Institutet, Stockholm, Sweden. [*]These authors contributed equally: [7]Current address; Department of Cell Biology and Neuroscience, University of California at Riverside, Riverside, California, USA .

Corresponding Author: Anna Krook
Department of Physiology and Pharmacology,
Section of Integrative Physiology,
Karolinska Institutet,
Biomedicum 4C
Solnavägen 9,
SE 171 77 Stockholm, Sweden
Phone: +46 (0) 8 524 878 24
Fax: +46 (0) 8 33 54 36
E-mail: anna.krook@ki.se

Supplementary Figure 1

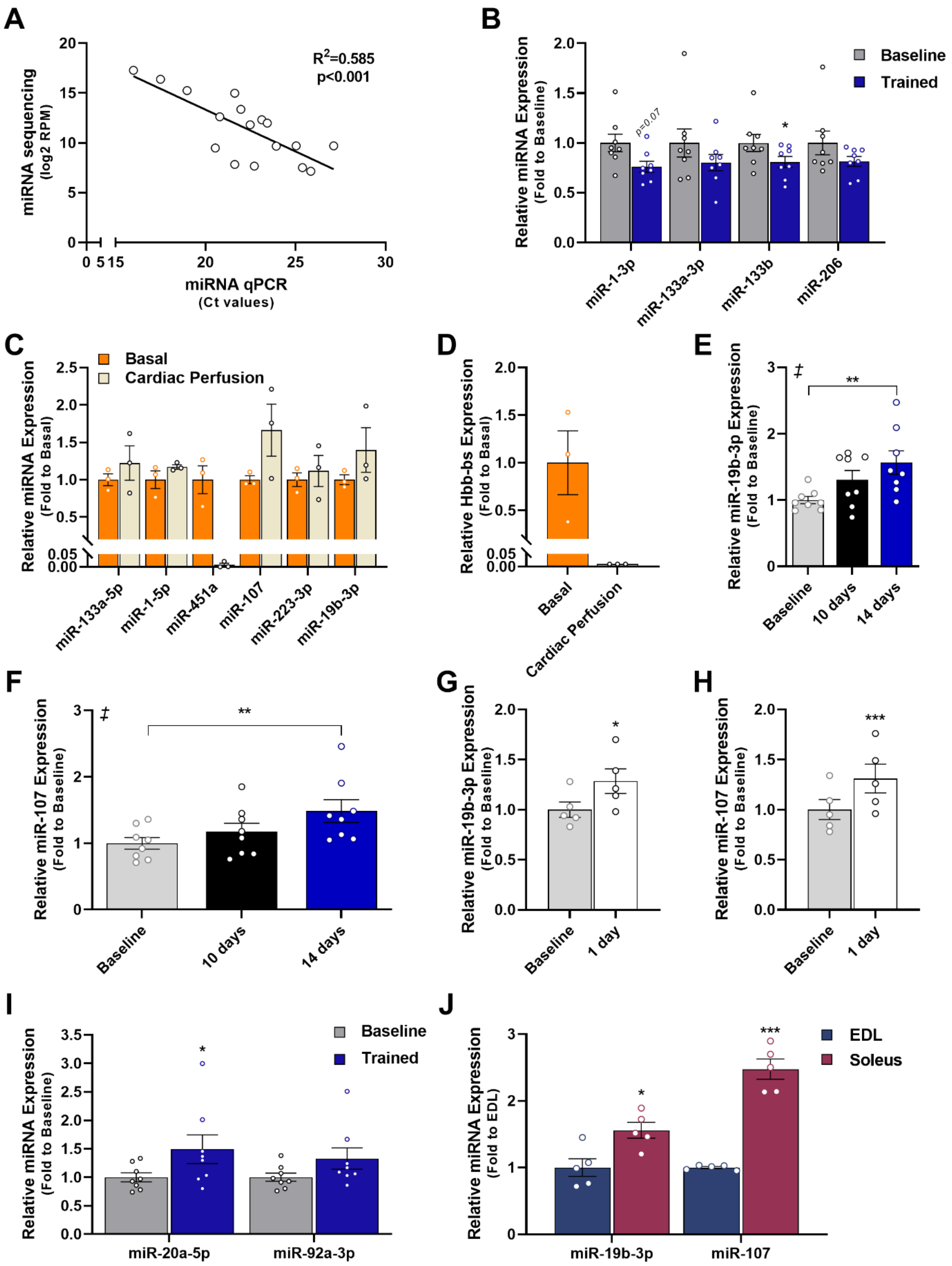

Supplementary Figure 1. Related to Figure 1. A) Correlation of short RNA sequencing and RT-qPCR data representing expression of 17 miRNAs in human skeletal muscle biopsies (p=0.0004, n=3 individuals). B) RT-qPCR determination of miRNA expression before (Baseline) and after aerobic exercise training (Trained) of myo-miRs (p=0.0371 for miR-133b, n=8 individuals). C) miRNA and D) Hbb-bs gene expression in soleus skeletal muscle of mice subjected to cardiac perfusion or not (Basal) determined by RT-qPCR (n=3 independent mouse samples). E-F) Expression of E) miR-19b-3p (p=0.0023 at day 14) and F) miR-107 (p=0.0053 at day 14) determined by RT-qPCR prior to exercise training (Baseline) or following 10 or 14 days of aerobic exercise training (n=8 individuals). G-H) Expression of G) miR-19b-3p (p=0.0129) and H) miR-107 (p=0.0001) prior to exercise training (Baseline) or 16 hours after the first bout of exercise (1 day) as determined by RT-qPCR (n=5 individuals). I) Expression of miR-20a-5p (p=0.0384) and miR-92a-3p prior to exercise training (Baseline) or following 14 days of aerobic exercise training (Trained) as determined by RT-qPCR (n=8 individuals). J) miR-19b-3p (p=0.0139) and miR-107 (p=0.0001) expression in mouse soleus and EDL skeletal muscle of mice (n=5 independent mouse samples). Data are mean ± SEM. miRNA expression was normalized to miR-186-5p in human and mouse samples. Gene expression in mouse skeletal muscle was normalized to *Tbp*. ‡ Endurance exercise training effect; and *p<0.05, **p<0.01, ***p<0.001 by two-tailed non-paired or paired Student's t-test or by repeated measures 1-way ANOVA with Dunnett's post hoc testing.

Supplementary Figure 2

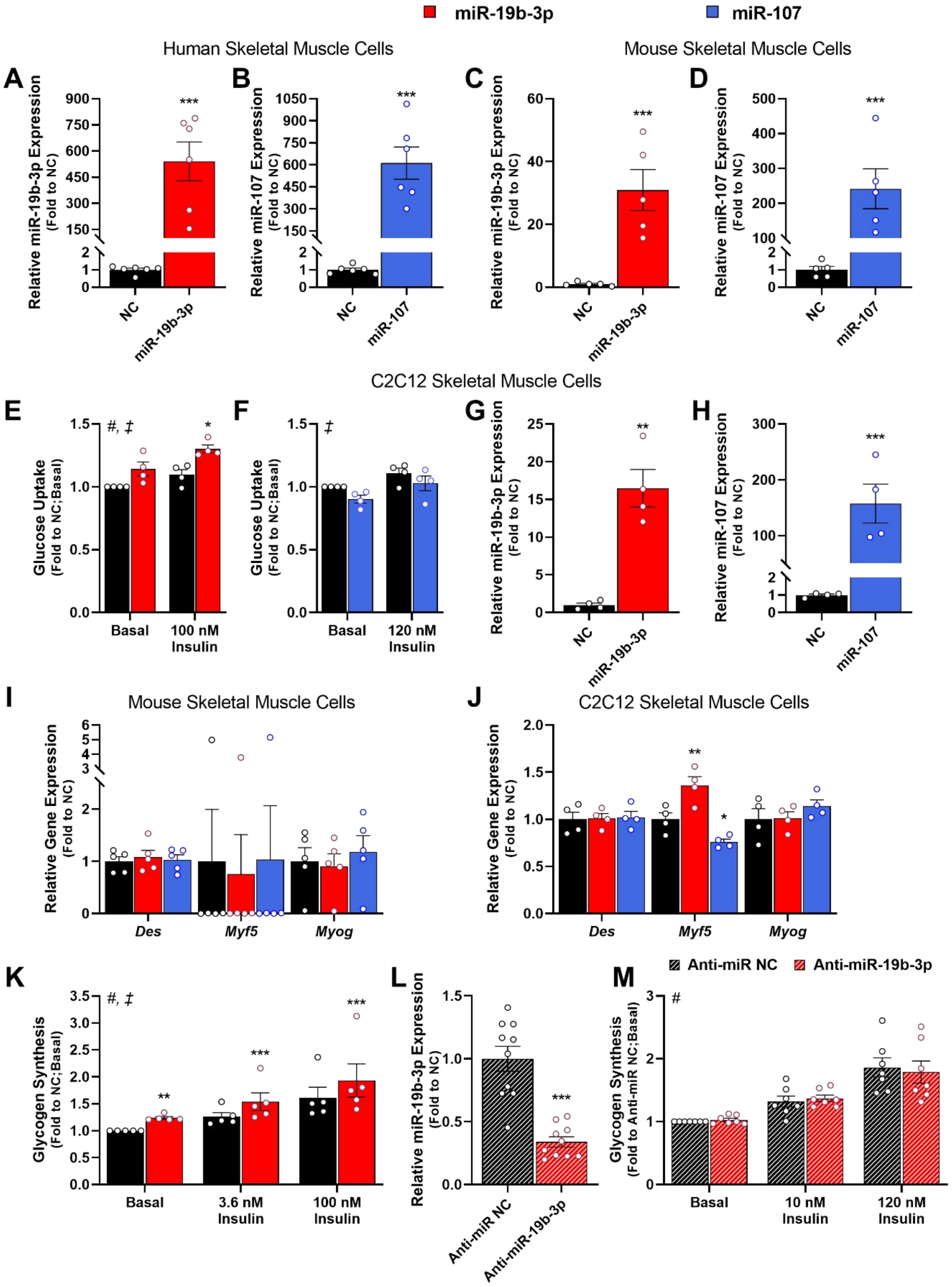

Supplementary Figure 2. Related to Figure 2, Myotubes were transfected with miR-19b-3p or miR-107 precursors or a negative control (NC) miRNA precursor. A-B) Overexpression of A) miR-19b-3p (p=0.0001) and B) miR-107 (p=0.0001) was assessed by RT-qPCR in human skeletal muscle cells (n=6 independent cell culture experiments). C-D) Overexpression of C) miR-19b-3p (p=0.0006) and D) miR-107 (p=0.0001) was assessed by RT-qPCR in mouse skeletal muscle cells (n=5 independent cell culture experiments). E-F) C2C12 myotubes were incubated in the absence (Basal) or presence of insulin (100 nM) to determine the effect of E) miR-19b-3p (p=0.049 following 100 nM insulin) and F) miR-107 overexpression on glucose uptake (n=4 independent cell culture experiments). G-H) Overexpression of G) miR-19b-3p (p=0.0028) and H) miR-107 (p=0.0002) was assessed by RT-qPCR in C2C12 skeletal muscle cells (n=4 independent cell culture experiments). I-J) Expression of *Des, Myf5,* and *Myog* was determined by RT-qPCR in I) mouse skeletal muscle cells or J) C2C12 skeletal muscle cells following overexpressing of either miR-19b-3p or miR-107 (n=4 independent cell culture experiments). (C2C12: miR-19b-3p p=0.0033 and miR-107 p=0.019 for *Myf5*). K) Mouse myotubes were incubated in the absence (Basal) or presence of insulin (3.6 nM or 100 nM) to determine the effects of miR-19b-3p overexpression on glucose incorporation into glycogen (n=5 independent cell culture experiments). (Basal p=0.0016, 3.6 nM insulin p=0.0006, and 100 nM insulin p=0.0003). L) Expression of *miR-19b-3p* was determined by RT-qPCR following transfection of human myotubes with either an anti-miR negative control (NC) or anti-miR-19b-3p inhibitor (p=0.0001, n=10 independent cell culture experiments). M) Human myotubes were incubated in the absence (Basal) or presence of insulin (10 nM or 120 nM) to determine the effects of miR-19b-3p inhibition on glucose incorporation into glycogen (n=7 independent cell culture experiments). Data are mean ± SEM. miRNA expression was normalized to miR-186-5p and gene expression to *Tbp*. # Treatment effect; ‡ miRNA effect; and *p<0.05, **p<0.01, ***p<0.001 by two-tailed paired Student's t-test or 2-way repeated measures ANOVA with Sidak's post hoc testing when appropriate.

Supplementary Figure 3

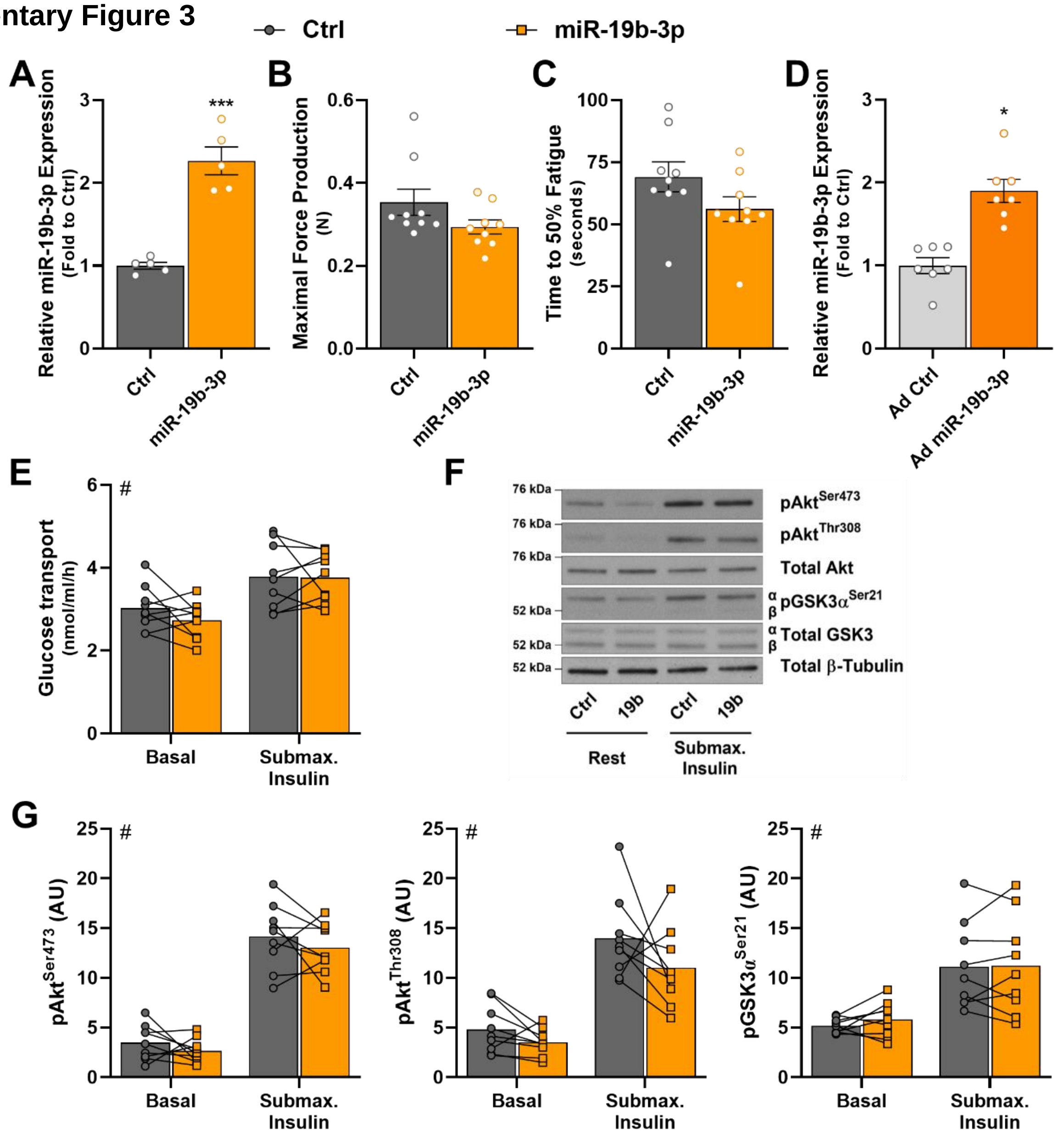

Supplementary Figure 3. Related to Figure 3. Mouse flexor digitorum brevis (FDB) skeletal muscles were electroporated with a control (Ctrl) or a miR-19b-3p-encoding plasmid and 1 week later muscles were subjected to electrical pulse-stimulated contraction or stimulated with insulin in vitro. A) Overexpression of miR-19b-3p was assessed by RT-qPCR in FDB skeletal muscle (p=0.001) (n=5 independent mouse samples). B) Maximal force (N) produced during electrical pulse-stimulated contraction in vitro by FDB muscles electroporated with control or miR-19b-3p-encoding plasmids (n=9 independent mouse samples). C) The time (in seconds) to reduce force production by 50% in FDB muscles during electrical pulse-stimulated contraction in vitro (n=9 independent mouse samples). D) RT-qPCR analysis of miR-19b-3p expression (normalized to miR-186-5p) in mouse FDB-transduced skeletal muscle 7-day post injection with a control (Ad Ctrl) or *mmu-miR-19b-1*-expressing adenovirus (Ad miR-19b-1) (p=0.016) (n=7 independent

mouse samples). E) FDB skeletal muscles electroporated with either control or miR-19b-3p-encoding plasmid were treated in vitro in absence (Basal) or presence of 3.6 nM insulin (Submax. Insulin) followed by measurement of glucose transport (n=9 independent mouse samples). F) Representative immunoblots of FDB skeletal muscle to determine the effects of miR-19b-3p overexpression on protein phosphorylation following treatment in absence or presence of 3.6 nM insulin (Submax. Insulin) in vitro. G) Quantification of (E) for phosphorylation of Akt (Thr^{308} and Ser^{473}) and GSK3α (Ser^{21}) (n=9 independent mouse samples). Data are mean ± SEM. miRNA expression was normalized to miR-186-5p. # Insulin effect by repeated measures 2-way ANOVA; and ***p<0.001 by two-tailed paired Student's t-test.

Supplementary Figure 4

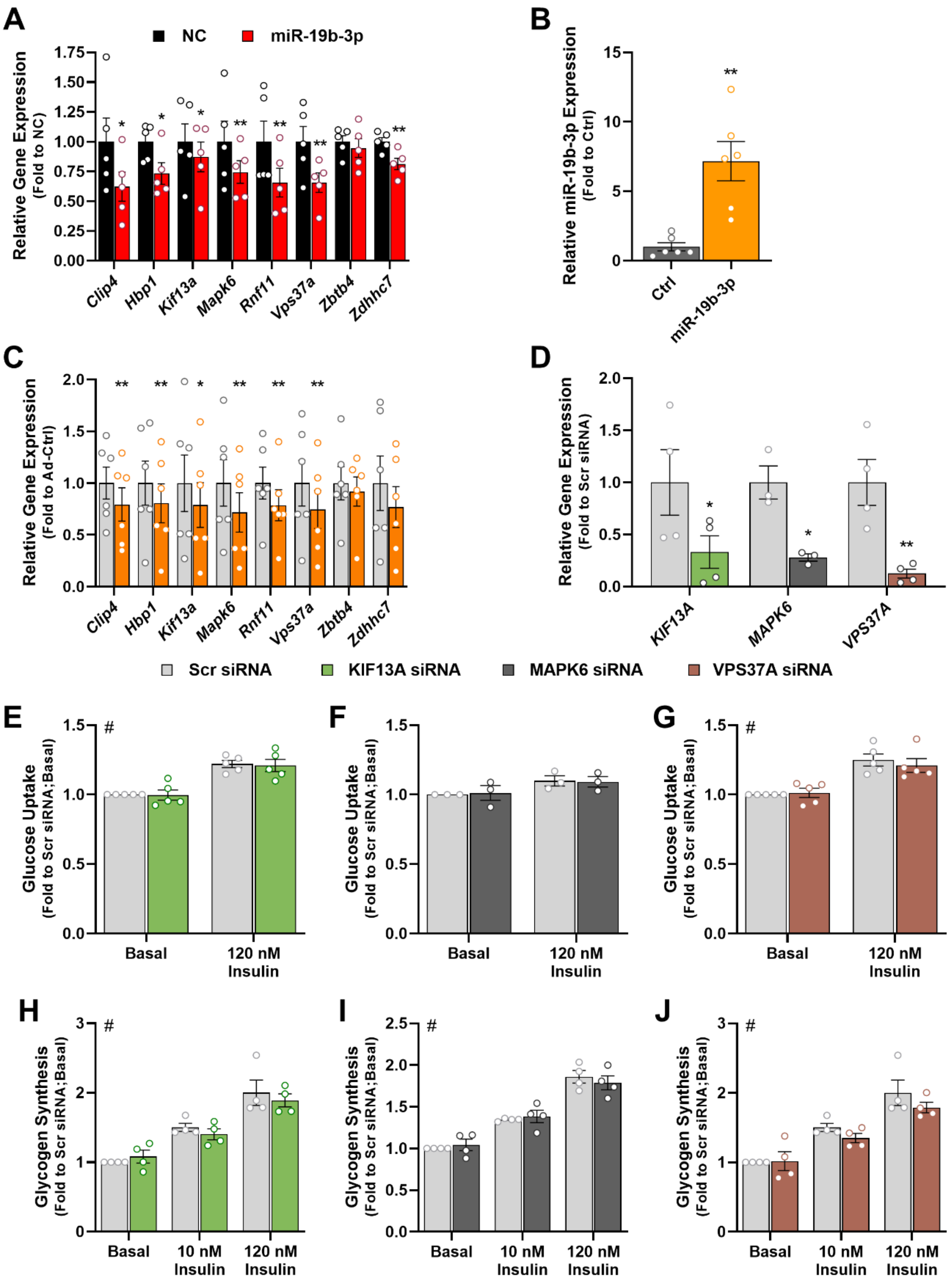

Supplementary Figure 4. Related to Figure 4. A) Mouse skeletal muscle myotubes were transfected with a negative control miRNA (NC) or miR-19b-3p mimics and gene expression of *Clip4* (p=0.0124), *Hbp1* (p=0.0318), *Kif13a* (p=0.0323), *Mapk6* (p=0.0086), *Rnf11* (p=0.008), *Vps37a* (p=0.0022), *Zbtb4*, and *Zdhhc7* (p=0.0027) was determined by RT-qPCR (n=5 independent cell culture experiments). Gene expression was normalized to *Tbp* expression. B) RT-qPCR analysis of miR-19b-3p expression (normalized to miR-186-5p) in mouse tibialis anterior skeletal muscle following electroporation with a control (Ctrl) or pri-miR-19b-3p-encoding plasmid (p=0.0055, n=6 independent mouse samples). C) RT-qPCR analysis of gene expression of *Clip4* (p=0.00015), *Hbp1* (p=0.0024), *Kif13a* (p=0.022), *Mapk6* (p=0.0016), *Rnf11* (p=0.0077), *Vps37a* (p=0.0043), *Zbtb4*, and *Zdhhc7* in mouse FDB-transduced skeletal muscle 7-day post injection with a control (Ad Ctrl) or *mmu-miR-19b-1*-expressing adenovirus (Ad miR-19b-1) (n=6 independent mouse samples) normalized to *Tbp* expression. D) Human skeletal muscle myotubes were transfected with a scrambled siRNA sequence (Scr siRNA) or gene specific siRNA sequences targeting KIF13A (n=4 independent cell culture experiments, p=0.0318), MAPK6 (n=3 independent cell culture experiments, p=0.0195), or VPS37A (n=4 independent cell culture experiments, p=0.0029), and thereafter gene expression was determined by RT-qPCR. Gene expression was normalized to the geometrical mean of *GUSB* and *TBP* expression. E-G) Human skeletal muscle myotubes were incubated in the absence (Basal) or presence of insulin (120 nM) to determine glucose uptake rate following silencing of E) KIF13A (n=5 independent cell culture experiments), F) MAPK6 (n=3 independent cell culture experiments), or G) VPS37A (n=5 independent cell culture experiments). H-J) Glycogen synthesis rate was determined in human skeletal muscle cells in the absence (Basal) or presence of insulin (10 nM or 120 nM) following silencing of H) KIF13A (n=4 independent cell culture experiments), I) MAPK6 (n=4 independent cell culture experiments), J) VPS37A (n=4 independent cell culture experiments). Data are mean ± SEM. # Contraction or Insulin effect; ‡ miRNA effect using repeated measures 2-way ANOVA; and *p<0.05, **p<0.01 and ***p<0.001 by two-tailed paired Student's t-test.

SUPPLEMENTARY TABLES

Supplementary Table 1: miRNAs with >100 reads per million (RPM) in at least half of the human skeletal muscle biopsies used for miRNA sequencing, related to figure 1.

miRNA	Pre Exercise Training (RPM)	Post Exercise Training (RPM)
hsa-miR-1-3p	208397 ± 61354	113142 ± 42193
hsa-miR-133a-3p	37577 ± 16582	40720 ± 3764
hsa-let-7c-5p	31870 ± 13260	35960 ± 10720
hsa-miR-133b	31238 ± 15654	33737 ± 2688
hsa-let-7b-5p	31047 ± 12910	35286 ± 10556
hsa-let-7a-5p	29562 ± 12453	33421 ± 9969
hsa-let-7f-5p	21434 ± 8186	24718 ± 6744
hsa-miR-29c-3p	19759 ± 5915	19675 ± 3012
hsa-miR-451a	18272 ± 7608	153160 ± 81194
hsa-miR-206	12371 ± 1978	8731 ± 1772
hsa-miR-23b-3p	11948 ± 823	15887 ± 2834
hsa-miR-24-3p	11488 ± 2580	5700 ± 743
hsa-miR-23a-3p	10891 ± 736	14213 ± 2422
hsa-miR-29a-3p	10389 ± 2306	9082 ± 1691
hsa-miR-26a-5p	10309 ± 1110	10733 ± 1477
hsa-miR-378a-3p	8861 ± 2238	3777 ± 3269
hsa-miR-22-5p	7947 ± 707	2377 ± 563
hsa-miR-378c	5864 ± 1642	2268 ± 2009
hsa-miR-101-3p	5482 ± 4092	11758 ± 2979
hsa-let-7i-5p	5223 ± 3089	9028 ± 1509
hsa-let-7g-5p	5040 ± 3081	8748 ± 1406
hsa-miR-29b-3p	4780 ± 998	6100 ± 678
hsa-let-7d-5p	4343 ± 2259	3153 ± 410
hsa-miR-133a-5p	4342 ± 487	2895 ± 2127
hsa-miR-126-3p	4068 ± 1768	5276 ± 1628
hsa-miR-499a-5p	3789 ± 765	3249 ± 123
hsa-miR-126-5p	3308 ± 696	2352 ± 395
hsa-miR-26b-5p	3166 ± 233	2570 ± 519
hsa-miR-21-5p	3098 ± 793	8360 ± 3289
hsa-miR-145-5p	2902 ± 2417	784 ± 178
hsa-miR-486-5p	2839 ± 582	3984 ± 535

hsa-miR-27b-3p	2210 ± 1044	3473 ± 698
hsa-miR-27a-3p	2107 ± 975	3359 ± 669
hsa-miR-30a-3p	2047 ± 15	1630 ± 152
hsa-miR-30e-3p	2025 ± 12	1613 ± 149
hsa-miR-16-5p	1808 ± 1122	1868 ± 120
hsa-miR-125b-5p	1783 ± 71	1750 ± 565
hsa-miR-199a-3p	1528 ± 401	1777 ± 392
hsa-miR-199b-3p	1527 ± 401	1777 ± 392
hsa-miR-378a-5p	1499 ± 608	1228 ± 179
hsa-miR-30b-5p	1423 ± 159	1484 ± 542
hsa-miR-22-3p	1195 ± 75	1634 ± 421
hsa-miR-95-3p	1191 ± 244	1477 ± 149
hsa-miR-30a-5p	1082 ± 563	2234 ± 130
hsa-miR-193b-3p	991 ± 301	955 ± 393
hsa-miR-143-3p	951 ± 256	1311 ± 213
hsa-miR-181a-5p	937 ± 108	967 ± 134
hsa-miR-365b-3p	904 ± 231	1261 ± 342
hsa-miR-365a-3p	904 ± 231	1261 ± 342
hsa-miR-196a-5p	889 ± 42	790 ± 182
hsa-miR-195-5p	884 ± 108	745 ± 107
hsa-miR-99a-5p	876 ± 134	1180 ± 157
hsa-miR-100-5p	868 ± 132	1171 ± 156
hsa-miR-15a-5p	815 ± 457	932 ± 113
hsa-miR-15b-5p	749 ± 210	882 ± 132
hsa-miR-92a-3p	694 ± 66	741 ± 210
hsa-miR-30d-5p	661 ± 306	2237 ± 88
hsa-miR-30c-5p	641 ± 111	999 ± 228
hsa-miR-128-3p	538 ± 207	412 ± 117
hsa-miR-221-3p	530 ± 254	875 ± 61
hsa-let-7e-5p	504 ± 90	563 ± 149
hsa-miR-98-5p	429 ± 284	664 ± 173
hsa-miR-378d	396 ± 64	200 ± 188
hsa-miR-144-3p	346 ± 77	464 ± 224
hsa-miR-181b-5p	344 ± 33	251 ± 42
hsa-miR-93-5p	332 ± 40	228 ± 53
hsa-miR-320a	309 ± 52	1002 ± 292
hsa-miR-320b	280 ± 48	894 ± 259
hsa-miR-140-3p	272 ± 26	539 ± 107

hsa-miR-30e-5p	264 ± 108	1375 ± 313
hsa-miR-191-5p	263 ± 15	478 ± 37
hsa-miR-196b-5p	249 ± 14	255 ± 55
hsa-miR-361-5p	229 ± 6	205 ± 27
hsa-miR-423-3p	213 ± 18	464 ± 73
hsa-miR-29c-5p	209 ± 29	633 ± 110
hsa-miR-20a-5p	206 ± 53	252 ± 52
hsa-miR-320d	197 ± 36	582 ± 159
hsa-miR-320e	194 ± 36	576 ± 157
hsa-miR-152-3p	184 ± 59	282 ± 81
hsa-miR-103a-3p	176 ± 63	1500 ± 166
hsa-miR-107	175 ± 63	1495 ± 167
hsa-let-7d-3p	171 ± 37	210 ± 22
hsa-miR-1297	160 ± 14	138 ± 32
hsa-miR-125a-5p	160 ± 64	186 ± 58
hsa-miR-193b-5p	141 ± 57	146 ± 35
hsa-miR-1827	138 ± 57	197 ± 84
hsa-miR-660-5p	133 ± 21	232 ± 80
hsa-miR-362-3p	126 ± 30	108 ± 27
hsa-miR-29a-5p	124 ± 47	148 ± 29
hsa-miR-199a-5p	120 ± 51	372 ± 106
hsa-miR-342-3p	118 ± 18	103 ± 19
hsa-miR-144-5p	134 ± 43	90 ± 46
hsa-miR-190a-5p	107 ± 34	92 ± 9
hsa-miR-374b-5p	96 ± 9	127 ± 9
hsa-miR-497-5p	89 ± 22	225 ± 48
hsa-miR-425-5p	83 ± 13	110 ± 12
hsa-miR-199b-5p	75 ± 32	129 ± 31
hsa-miR-1-5p	71 ± 42	337 ± 129
hsa-miR-484	60 ± 5	170 ± 6
hsa-miR-130a-3p	57 ± 16	167 ± 13
hsa-miR-223-3p	44 ± 34	325 ± 15
hsa-miR-19b-3p	38 ± 8	248 ± 52

Short RNA sequencing of human skeletal muscle biopsies at baseline (pre exercise training) and following two weeks of endurance exercise training (post exercise training) was utilized to determine miRNA abundance in skeletal muscle biopsies (reads per million (RPM) > 100 in at least three samples). Results are mean ± SEM for three biopsies per condition.

Supplementary Table 2: Quantification of total and phosphorylated proteins following miR-19b-3p overexpression in human skeletal muscle cells, related to figure 2.

	**Basal**		**10 nM Insulin**		**120 nM Insulin**		**ANOVA Summary**
	NC	***miR-19b-3p***	***NC***	***miR-19b-3p***	***NC***	***miR-19b-3p***	
pTBC1D1 (S237)	1.00±0.28	1.57±0.46	1.26±0.33	1.76±0.28	1.09±0.20	1.84±0.53	‡
Tot. TBC1D1	1.00±0.23	1.44±0.31** (p=0.0036)	1.10±0.27	1.50±0.27** (p=0.0069)	1.14±0.26	1.38±0.31	‡
pAS160 (T642)	1.00±0.21	2.50±0.53** (p=0.0080)	3.54±0.82	6.11±0.61*** (p=0.0001)	6.81±0.65	7.59±0.64	#, ‡, $
Tot. AS160	1.00±0.21	1.08±0.17	1.23±0.13	1.45±0.17	1.29±0.19	1.52±0.22	
pGS (S641)	1.00±0.18	1.02±0.19	0.98±0.19	1.07±0.17	1.05±0.21	0.99±0.16	
Tot. GS	1.00±0.11	0.95±0.15	1.14±0.13	1.27±0.14	1.66±0.20	1.55±0.17	#
pAkt (S473)	1.00±0.59	1.76±1.78	22.16±6.43	66.06±15.56** (p=0.0063)	176.43±21.27	200.73±19.88	#
pAkt (T308)	1.00±0.59	2.67±1.43	13.48±4.26	40.27±7.26**	95.79±6.80	101.31±5.66	#, $
Tot. Akt	1.00±0.07	1.01±0.04	0.99±0.04	1.04±0.02	0.96±0.03	1.04±0.03	
pGSK3α (S21)	1.00±0.10	1.54±0.18* (p=0.0237)	1.48±0.21	2.53±0.20*** (p=0.0002)	2.52±0.34	2.95±0.33	#, ‡

Tot. GSK3α	1.00±0.11	1.41±0.18** (p=0.0038)	1.09±0.10	1.51±0.09** (p=0.0036)	1.00±0.13	1.25±0.17	‡
pGSK3β (S9)	1.00±0.12	0.89±0.14	1.62±0.24	1.79±0.22	2.45±0.30	2.28±0.31	#
Tot. GSK3β	1.00±0.12	0.98±0.13	1.03±0.08	1.01±0.08	0.95±0.08	0.96±0.11	

Myotubes were transfected with miRNA precursors for either miR-19b-3p or with a negative control (NC) and were thereafter incubated in absence (Basal) or presence of insulin (10 nM or 120 nM) for 10 minutes. Phosphorylation and total protein abundance were determined by Western blot. Results are mean ± SEM expressed as fold to NC for n=6 independent cell culture experiments. # Treatment (Insulin) effect; ‡ miRNA (miR-19b-3p) effect; $ Interaction; and *p<0.05, **p<0.01, ***p<0.001 comparing Neg. Control vs miR-19b-3p by a 2-way repeated measures ANOVA with Sidak's post hoc testing.

.

Supplementary Table 3: Gene ontology analysis following miR-19b-3p overexpression in human skeletal muscle cells, related to figure 4.

Gene Ontology ID	Description	Gene Ratio	Background Ratio	p-value	p.adjust
GO:0030198	extracellular matrix organization	28/590	327/16303	2.33E-05	4.02E-02
GO:0043062	extracellular structure organization	28/590	328/16303	2.46E-05	4.02E-02
GO:0016125	sterol metabolic process	17/590	151/16303	3.36E-05	4.02E-02
GO:0008299	isoprenoid biosynthetic process	7/590	27/16303	3.70E-05	4.02E-02
GO:0061082	myeloid leukocyte cytokine production	6/590	22/16303	9.95E-05	5.42E-02
GO:0008203	cholesterol metabolic process	15/590	134/16303	1.03E-04	5.42E-02
GO:0008202	steroid metabolic process	25/590	304/16303	1.21E-04	5.42E-02
GO:0031346	positive regulation of cell projection organization	26/590	323/16303	1.26E-04	5.42E-02
GO:1902652	secondary alcohol metabolic process	15/590	138/16303	1.43E-04	5.42E-02
GO:0006024	glycosaminoglycan biosynthetic process	13/590	109/16303	1.55E-04	5.42E-02
GO:1903034	regulation of response to wounding	16/590	155/16303	1.60E-04	5.42E-02
GO:1901615	organic hydroxy compound metabolic process	34/590	482/16303	1.67E-04	5.42E-02
GO:0006023	aminoglycan biosynthetic process	13/590	110/16303	1.70E-04	5.42E-02
GO:0016266	O-glycan processing	9/590	57/16303	1.91E-04	5.42E-02
GO:0061564	axon development	33/590	466/16303	1.93E-04	5.42E-02

GO:0006066	alcohol metabolic process	25/590	314/16303	2.00E-04	5.42E-02
GO:0045671	negative regulation of osteoclast differentiation	6/590	26/16303	2.71E-04	6.94E-02
GO:0045670	regulation of osteoclast differentiation	9/590	62/16303	3.67E-04	8.85E-02
GO:0006909	phagocytosis	19/590	219/16303	3.96E-04	9.05E-02
GO:0061081	positive regulation of myeloid leukocyte cytokine production involved in immune response	4/590	11/16303	4.57E-04	9.94E-02

Myotubes were transfected with miRNA precursors for either miR-19b-3p or with a negative control (NC) and gene expression was determined by microarray analysis. miR-19b-3p-regulated gene ontologies (p.adjust<0.1) are reported (Benjamini Hochberg adjustment).

Supplementary Table 4: miR-19b-3p predicted targets downregulated by miR-19b-3p overexpression or aerobic exercise training, related to figure 4.

Gene Symbol	Log2 FC (19b-3p OE)	p-value (19b-3p OE)	Number of hits in exercise studies
RNF11	-2.10	4.77E-05	2
CLIP4	-1.07	1.29E-03	2
RRM2B	-1.06	1.58E-04	2
MAPK6	-0.99	2.45E-04	3
TPCN1	-0.96	4.37E-04	2
VPS37A	-0.92	1.82E-03	3
ZDHHC7	-0.91	1.17E-03	2
CACUL1	-0.87	4.61E-04	2
EPG5	-0.85	4.21E-04	2
HBP1	-0.85	6.60E-04	2
PHF20	-0.84	6.18E-04	3
FBXO32	-0.80	8.13E-04	2
ZBTB4	-0.80	3.12E-03	3
JUND	-0.80	5.79E-03	3
KIF13A	-0.76	2.57E-03	3
RNF144B	-0.75	3.09E-03	3
CLIP1	-0.70	3.08E-03	3
JMY	-0.68	3.58E-03	2
MDFIC	-0.66	2.27E-03	2
TRIM23	-0.63	6.39E-03	2
TMEM47	-0.63	2.34E-03	2
DEK	-0.63	2.29E-03	2
PNRC1	-0.63	5.42E-03	3
RAB21	-0.62	6.40E-03	3
YIPF6	-0.62	2.31E-03	3

DLG5	-0.61	6.70E-03	2

Myotubes were transfected with miRNA precursors for either miR-19b-3p or with a negative control (NC) and gene expression was determined by microarray analysis. miR-19b-3p-regulated genes were overlapped with genes altered in publicly available microarray studies representing aerobic exercise training [8, 15, 16]. miR-19b-3p predicted targets (TargetScan or microRNA.org) with similar fold change directionality following miR-19b-3p overexpression and in at least two of the exercise training studies are presented (p-values from two-sided moderated t-statistic, no adjustment).

Supplementary Table 5: TaqMan primers (Thermo Fisher Scientific) used for miRNA real-time qPCR.

miRNA	TaqMan Advanced miRNA Primer Assay ID
hsa-miR-1-3p	477820_mir
hsa-miR-1-5p	478726_mir
hsa-miR-16-5p	477860_mir
hsa-miR-19b-3p	478264_mir
hsa-miR-21-5p	477973_mir
hsa-miR-22-5p	477987_mir
hsa-miR-24-3p	477992_mir
hsa-miR-29a-3p	478587_mir
hsa-miR-29b-3p	478369_mir
hsa-miR-29c-3p	479229_mir
hsa-miR-30b-5p	478007_mir
hsa-miR-30e-5p	479235_mir
hsa-miR-103a-3p	478253_mir
hsa-miR-107	478254_mir
hsa-miR-125b-5p	477885_mir
hsa-miR-126-5p	477888_mir
hsa-miR-133a-3p	478511_mir
hsa-miR-133a-5p	478706_mir
hsa-miR-133b	480871_mir
hsa-miR-143-3p	477912_mir
hsa-miR-181a-5p	477857_mir
hsa-miR-186-5p	477940_mir
hsa-miR-191-5p	477952_mir
hsa-miR-195-5p	477957_mir
hsa-miR-206	477968_mir
hsa-miR-223-3p	477983_mir
hsa-miR-320a	478594_mir
hsa-miR-378a-3p	478349_mir
hsa-miR-378c	478864_mir
hsa-miR-451a	478107_mir
hsa-miR-486-5p	478128_mir

Supplementary Table 6: Primers used for real-time qPCR.

Target Gene	Forward Primer Sequence	Reverse Primer Sequence
**Human sequences**		
CLIP4	ATGTCACTGGCAAGGCAATG	TCATCCAGTTCAATGCCAGC
DES	CTGGAGCGCAGAATTGAATC	GGCAGTGAGGTCTGGCTTAG
GUSB	CAGAGCGAGTATGGAGCAGA	ACTCTCGTCGGTGACTGTTC
HBP1	GACACCCCGATGCCATTAAT	CCACAAGACAAAGATGCACGA
KIF13A	AAGACTGTCTCCGATGGGC	GAACAAAGACGCCAAGCTGT
MAPK6	CATTGACATGTGGGCTGCAG	GCTCCTGACGATCTTCCTCA
MYF5	CCACCTCCAACTGCTCTGAT	GCAATCCAAGCTGGATAAGG
MYOG	GCTCAGCTCCCTCAACCA	GCTGTGAGAGCTGCATTCG
PTEN	TCTGCCATCTCTCTCCTCCT	CGCCTTCAAGTCTTTCTGCA
RNF11	TTCACGAGTCTCAGTCCGAC	GTGTTGGGTGGTAGACTGGA
TBP	AACAACAGCCTGCCACCTTA	GCCATAAGGCATCATTGGAC
VPS37A	TGCCACTGTAGAAGAGCCAA	TCCCATTCCTCTCTTGGCAG
ZBTB4	GAAGTACCCCTGCCGCTATT	GCTGGTGGGTCTTCAGGTTA
ZDHHC7	TCCTGAAGAAACGGCACAAA	AGCCAGGAGAGGATGATGC
**Mouse sequences**		
Clip4	AGTTGCTCTCCGAGATATGGA	TCTGAAAGCGTATCCAGGGA
Des	ATGGCCTTGGATGTGGAGAT	GGCTGGTTTCTCGGAAGTTG
Hbb_bs	TGAGCTCCACTGTGACAAGC	GTGAAATCCTTGCCCAGGT
Hbp1	TGGACACCCTGATGCCATTA	CACAAGACAGAGATGCACGG
Kif13a	GACATCACTCTCACGCCCAA	CCCCATAAGATCCTGTCGCC
Mapk6	CACATGAACTTGAACAGATGCAG	CAGTGCTTCTCGGCTGATCC
Myf5	TGAGGGAACAGGTGGAGAAC	TGTTCTTTCGGGACCAGACA
Myog	CCAGTGAATGCAACTCCCAC	AGATTGTGGGCGTCTGTAGG
Pten	ACAAAGACAAGGCCAACCGAT	TGAACTGCTAGCCTCTGGAT
Rnf11	GGCCTTATACAGCATCTGCCT	CATGCACGGCAGAAATCGAA
Tbp	CCTTGTACCCTTCACCAATGAC	ACAGCCAAGATTCACGGTAGA

Vps37a	ATCCTCCAGTTTTAGCTCCTACTTC	AGCTTCTGGTGGAGGATATGG
Zbtb4	CTGTGAGAAGGTGTTTGCCC	TCGCTGGTGGGTCTTCAG
Zdhhc7	CAAGTGCTCCCATGATGCTT	GAGGACGAAGAGGATGCTGA

Supplementary Table 7: Antibodies used.

TARGET	SOURCE	IDENTIFIER	DILUTION
Acetyl-CoA Carboxylase	Cell Signaling	3676	1/1000
Phospho-Acetyl-CoA Carboxylase (Ser79)	Cell Signaling	3661	1/1000
Akt	Cell Signaling	9272	1/1000
Phospho-Akt (Ser473)	Cell Signaling	9271	1/1000
Phospho-Akt (Thr308)	Cell Signaling	4056	1/1000
AMPKα (α1 and α2)	Cell Signaling	2532	1/1000
AMPKα (Thr172)	Cell Signaling	2531	1/1000
AS160	Abcam	ab24469	1/1000
Phospho-AS160 (Ser588)	Cell Signaling	8730	1/1000
Phospho-AS160 (Thr642)	Cell Signaling	8881	1/1000
β-actin	Sigma	A5441	1/5000
β-Tubulin	Cell Signaling	2128	1/1000
GLUT4	Millipore	07-1404	1/1000
Glycogen Synthase	Cell Signaling	3893	1/1000
Phospho-Glycogen Synthase (Ser641)	Cell Signaling	3891	1/1000
Glycogen synthase kinase 3 α/β	Cell Signaling	5676	1/1000
Phospho-Glycogen synthase kinase 3 α/β (Ser21/9)	Cell Signaling	9331	1/1000
MYH1/2	Santa Cruz Biotech.	sc-53088	1/500
Total OXPHOS Rodent WB Antibody Cocktail	Abcam	ab110413	1/1000
TBC1D1	Cell Signaling	4629	1/1000
Phospho-TBC1D1 (Ser237 or Ser231)	Millipore	07-2268	1/1000
Phospho-TBC1D1 (Ser700)	Cell Signaling	6929	1/1000
