# Supplementary material for: Endurance exercise training-responsive miR-19b-3p improves skeletal muscle glucose metabolism
Source: Nat Commun. 2021 Oct 12;12:5948. doi: 10.1038/s41467-021-26095-0 (PMC8511155; doi:10.1038/s41467-021-26095-0)
Supplement: Supplementary file 2 — Description of Additional Supplementary Information [file 41467_2021_26095_MOESM2_ESM.pdf]

Description of Additional Supplementary Information

Title: Supplementary Data 1: Transcriptomic analysis of gene expression alterations induced by miR-19b-3p overexpression in human skeletal muscle cells, related to Figure 4.

Description: Myotubes were transfected with miRNA precursors for either miR-19b-3p or a negative control (NC) and gene expression was determined by microarray analysis. File includes one tab with expression of all genes and one tab with genes considered to be altered by miR-19b-3p (p-value<0.01 and at least 1.5 fold; False Discovery Rate adjustment method, two-sided moderated t-statistics).
