# Supplementary material for: Endurance exercise training-responsive miR-19b-3p improves skeletal muscle glucose metabolism
Source: Nat Commun. 2021 Oct 12;12:5948. doi: 10.1038/s41467-021-26095-0 (PMC8511155; doi:10.1038/s41467-021-26095-0)
Supplement: Supplementary file 4 — Reporting summary [file 41467_2021_26095_MOESM4_ESM.pdf]

nature research

Corresponding author(s): Anna Krook
Last updated by author(s): Jul 21, 2021

Reporting Summary

Nature Research wishes to improve the reproducibility of the work that we publish. This form provides structure for consistency and transparency in reporting. For further information on Nature Research policies, see Authors & Referees and the Editorial Policy Checklist .

Statistics

For all statistical analyses, confirm that the following items are present in the figure legend, table legend, main text, or Methods section.

n/a	Confirmed	
[ ]	[x]	The exact sample size (*n*) for each experimental group/condition, given as a discrete number and unit of measurement
[ ]	[x]	A statement on whether measurements were taken from distinct samples or whether the same sample was measured repeatedly
[ ]	[x]	The statistical test(s) used AND whether they are one- or two-sided<br>*Only common tests should be described solely by name; describe more complex techniques in the Methods section.*
[ ]	[x]	A description of all covariates tested
[ ]	[x]	A description of any assumptions or corrections, such as tests of normality and adjustment for multiple comparisons
[ ]	[x]	A full description of the statistical parameters including central tendency (e.g. means) or other basic estimates (e.g. regression coefficient) AND variation (e.g. standard deviation) or associated estimates of uncertainty (e.g. confidence intervals)
[ ]	[x]	For null hypothesis testing, the test statistic (e.g. *F*, *t*, *r*) with confidence intervals, effect sizes, degrees of freedom and *P* value noted<br>*Give P values as exact values whenever suitable.*
[x]	[ ]	For Bayesian analysis, information on the choice of priors and Markov chain Monte Carlo settings
[x]	[ ]	For hierarchical and complex designs, identification of the appropriate level for tests and full reporting of outcomes
[ ]	[x]	Estimates of effect sizes (e.g. Cohen's *d*, Pearson's *r*), indicating how they were calculated

Our web collection on statistics for biologists contains articles on many of the points above.

Software and code

Policy information about availability of computer code

Data collection	Short RNA-seq: Illumina Genome Analyzer<br>Microarray transcriptome: Gene Titan Instrument<br>RT-qPCR: Step-One Software (v. 2.3)
Data analysis	Short RNA-seq: R software (v 3.4.2), FastX package, Bowtie2 package, featureCounts package (v 1.22.2), edgeR glmQLFit/glmQLFTest framework<br>Microarray transcriptome: R software (v 3.4.2), Oligo package (v 1.40.2), WGCNA package (v 3.5), limma package (v 3.32.10), clusterProfiler (v 3.4.4)<br>RT-qPCR: Step-One Software (v. 2.3)<br>GraphPad Prism (v. 8)<br>Labchart software (v. 7)<br>Quantity One (v. 4.6.6)

For manuscripts utilizing custom algorithms or software that are central to the research but not yet described in published literature, software must be made available to editors/reviewers. We strongly encourage code deposition in a community repository (e.g. GitHub). See the Nature Research guidelines for submitting code & software for further information.

Data

Policy information about availability of data

All manuscripts must include a data availability statement. This statement should provide the following information, where applicable:

- Accession codes, unique identifiers, or web links for publicly available datasets
- A list of figures that have associated raw data
- A description of any restrictions on data availability

Short RNA-seq data generated in this study have been deposited in the NCBI's GEO database under accession number GSE127187. The GEO accession number for

the gene array-generated transcriptomic data reported in this paper is GSE126187. Raw data of all figures and uncropped versions of blots presented in the figures are provided as a Source Data file.
The NCBI's GEO accession number for published microarray data analyzed in this paper are: GSE356618 (PMID: 18716044); GSE2753616 (PMID: 21909251); and GSE910315 (PMID: 20930125).
R scripts for the main steps of analysis are available from the corresponding author on reasonable request.

Field-specific reporting

Please select the one below that is the best fit for your research. If you are not sure, read the appropriate sections before making your selection.

- [x] Life sciences
- [ ] Behavioural & social sciences
- [ ] Ecological, evolutionary & environmental sciences

For a reference copy of the document with all sections, see nature.com/documents/nr-reporting-summary-flat.pdf

Life sciences study design

All studies must disclose on these points even when the disclosure is negative.

Sample size	Sample sizes were chosen based on previous experience from work with human, cell and mouse studies (PMID: 21873433; PMID: 28404597; PMID: 34131782).
Data exclusions	No data was excluded.
Replication	Data from human cells were obtained from cells obtained from at least three different donors and were based on data acquired from at least two independent experiments. N-sizes for mouse cells indicates number of replicated experiments (minimum 4 independent experiments).<br>Animal experiment data were based on 2 independent experiments.<br>All the results have been replicated in the independent repeated experiments.
Randomization	For animal experiments, mice are their own control (as one leg is control and the other overexpressing miRNA). Mice were randomly allocated to basal or contraction conditions.
Blinding	The investigators were blinded to group allocations during data collection and analysis.

Reporting for specific materials, systems and methods

We require information from authors about some types of materials, experimental systems and methods used in many studies. Here, indicate whether each material, system or method listed is relevant to your study. If you are not sure if a list item applies to your research, read the appropriate section before selecting a response.

Materials & experimental systems

n/a	Involved in the study	
[ ]	[x]	Antibodies
[ ]	[x]	Eukaryotic cell lines
[x]	[ ]	Palaeontology
[ ]	[x]	Animals and other organisms
[ ]	[x]	Human research participants
[x]	[ ]	Clinical data

Methods

n/a	Involved in the study	
[x]	[ ]	ChIP-seq
[x]	[ ]	Flow cytometry
[x]	[ ]	MRI-based neuroimaging

Antibodies

Antibodies used

TARGET / SOURCE / IDENTIFIER / DILUTION
Acetyl-CoA Carboxylase Cell Signaling 3676 1/1000
Phospho-Acetyl-CoA Carboxylase (Ser79) Cell Signaling 3661 1/1000
Akt Cell Signaling 9272 1/1000
Phospho-Akt (Ser473) Cell Signaling 9271 1/1000
Phospho-Akt (Thr308) Cell Signaling 4056 1/1000
AMPKα (α1 and α2) Cell Signaling 2532 1/1000
AMPKα (Thr172) Cell Signaling 2531 1/1000
AS160 Abcam ab24469 1/1000
Phospho-AS160 (Ser588) Cell Signaling 8730 1/1000
Phospho-AS160 (Thr642) Cell Signaling 8881 1/1000
β-actin Sigma A5441 1/5000

	β-Tubulin Cell Signaling 2128 1/1000<br>GLUT4 Millipore 07-1404 1/1000<br>Glycogen Synthase Cell Signaling 3893 1/1000<br>Phospho-Glycogen Synthase (Ser641) Cell Signaling 3891 1/1000<br>Glycogen synthase kinase 3 α/β Cell Signaling 5676 1/1000<br>Phospho-Glycogen synthase kinase 3 α/β (Ser21/9) Cell Signaling 9331 1/1000<br>MYH1/2 Santa Cruz Biotech. sc-53088 1/500<br>Total OXPHOS Rodent WB Antibody Cocktail Abcam ab110413 1/1000<br>TBC1D1 Cell Signaling 4629 1/1000<br>Phospho-TBC1D1 (Ser237 or Ser231) Millipore 07-2268 1/1000<br>Phospho-TBC1D1 (Ser700) Cell Signaling 6929 1/1000
Validation	Validation of the above mentioned antibodies as well as relevant citations are available on the manufacturer's website.

Eukaryotic cell lines

Policy information about cell lines

Cell line source(s)	C2C12 (ATCC)
Authentication	None of the cells were authenticated in house, but acquired directly from ATCC.
Mycoplasma contamination	Cells were tested negative for mycoplasma.
Commonly misidentified lines (See ICLAC register)	None of the cells used in this study are listed in ICLAC.

Animals and other organisms

Policy information about studies involving animals; ARRIVE guidelines recommended for reporting animal research

Laboratory animals	C57Bl/6J (Charles River) 15-week old males and 3-week old males and females
Wild animals	No wild animals were used
Field-collected samples	The study did not involve field-collected samples.
Ethics oversight	All animal experiments and protocols were approved by the Regional Animal Ethical Committee (Stockholm, Sweden).

Note that full information on the approval of the study protocol must also be provided in the manuscript.

Human research participants

Policy information about studies involving human research participants

Population characteristics	Two separate cohorts were used.<br>Eight healthy, sedentary men (mean age: 23) volunteered to participate in the first study (PMID: 21360670).<br>A subset of 5 of 8 healthy, sedentary men (mean age: 24) volunteered to participate in the second study (PMID: 20308248).<br>Subjects who volunteered were healthy and willing and able to perform cycle exercise and were self selected in this way. All subjects are compared to their own baseline thus minimizing effects of prior training. All subjects studied were male and results may be different in a female cohort.
Recruitment	Subjects responded to local advert.
Ethics oversight	All participants provided written informed consent and the research ethical committee at Dublin City University approved the study protocols and experimental procedures were conducted according to the Declaration of Helsinki.

Note that full information on the approval of the study protocol must also be provided in the manuscript.
